# Urban Hyperendemicity and Emerging Coastal Per‐Capita Risk of Dengue in Bangladesh, 2021–2025: A Nationwide Spatiotemporal Ecological Analysis

**DOI:** 10.1155/jotm/3012361

**Published:** 2026-08-07

**Authors:** Pratyay Hasan, Tazdin Delwar Khan, Mohammad Saiful Islam, Mohd. Arifuzzaman, Mohammad Emdadul Haque

**Affiliations:** ^1^ Emergency Department, Dhaka Dental College & Hospital, Dhaka, Bangladesh; ^2^ Department of Cardiac Anesthesiology, Ibrahim Cardiac Hospital & Research Institute, Dhaka, Bangladesh; ^3^ Department of Public Health, Directorate General of Health Services (DGHS) and Adjunct Faculty, Bangladesh Open University, Gazipur, Bangladesh; ^4^ Department of Public Health, North Western University, Khulna, Bangladesh; ^5^ Department of Oral and Maxillofacial Surgery, Bangabandhu Sheikh Mujib Medical University (BSMMU), Dhaka, Bangladesh, bsmmu.edu.bd

**Keywords:** Bangladesh, coastal risk, dengue, hotspots, SaTScan, spatial analysis, surveillance, urban epidemiology

## Abstract

**Background:**

Dengue has shifted from Dhaka‐centric to nationwide in Bangladesh.

**Aim:**

To quantify spatiotemporal patterns and identify high‐risk clusters across all 64 districts from 2021 to 2025, and, extending previous reports with data from 2025, this study aims to observe an ongoing shift in per‐capita risk hotspots.

**Methods:**

Retrospective ecological study of the Directorate General of Health Services (DGHS) aggregate yearly data on suspected and laboratory‐confirmed dengue admissions (*n* = 637,626 cases). Incidence per 100,000 population was calculated using 2022 census projections with 1.12% annual growth. Global (Moran’s I) and local (Getis‐Ord *Gi∗*) spatial autocorrelation and prospective space–time scan statistics (SaTScan) were applied.

**Results:**

National incidence: 76.4/100,000/year. 2025 rate‐adjusted Moran’s I = 0.328 (*p* = 0.001). Raw‐case hotspots: Greater Dhaka; population‐adjusted hotspots: Jhalokathi, Barguna (coastal). High‐rate space–time cluster (2024–2025): Dhaka, Manikganj (RR = 3.13, *p* = 0.001). Dhaka O/E = 4.31 (RR = 6.38). Two low‐rate clusters (north, southeast).

**Conclusion:**

Urban hyperendemicity, characterized by a high case volume, coupled with the emerging coastal per‐capita risk, necessitates tailored interventions. Intensive vector control is essential in Dhaka, while early‐warning surveillance should be implemented in coastal districts.

## 1. Introduction

Dengue fever, a viral infection transmitted primarily by *Aedes aegypti* and *Aedes albopictus* mosquitoes [1], represents a substantial global public health concern, particularly across tropical and subtropical regions [2]. In Bangladesh, the disease has posed a significant and recurring challenge, burdening the healthcare system and resulting in high morbidity and mortality [1, 3]. While the country first encountered dengue in 1964–1965, known then as “Dhaka Fever,” it emerged as a large‐scale epidemic in 2000 [4]. Since that time, dengue has remained a persistent health issue in the country [5].

Historically, Dhaka city has been the most recognized endemic zone and the epicenter of outbreaks [3]. In the large 2019 outbreak, Dhaka accounted for over half (51%) of the reported cases nationwide, and over 83% of cases in 2021 were concentrated in the capital [6]. This intense concentration of risk in Dhaka is linked to its dense population [7] and specific urban environmental components (UECs) [7]. Studies show that the presence of urban heat islands (UHIs), characterized by higher land surface temperatures (27°C–32°C) and dense, unplanned built‐up areas, creates favorable conditions for *Aedes aegypti* breeding and transmission [7].

However, recent years have marked a critical transition in disease dynamics, characterized by increasingly large outbreaks and a wider geographical spread [3]. The focus of the endemic has “radiated to various corners of the country” [5]. The year 2023 witnessed the most significant outbreak on record, with 321,179 reported cases, a five‐fold increase compared to 2022 [6]. Crucially, the proportional burden on Dhaka city decreased, dropping from 51% in 2019 to 34% in 2023 [6]. The disease achieved complete geographical coverage across all 64 districts in July 2023, peaking earlier than previously observed [6].

This spatial shift necessitates a renewed evaluation of the spatiotemporal patterns [3]. Prior research often focused narrowly on urban settings like Dhaka [8–10]. While previous studies documented geographic expansion (e.g., Hossain et al., 2024, identified stable southern hotspots from 2019–2023, including Bagerhat, Jhalokathi, and Patuakhali in 2022‐2023), this work advances an understanding by providing updated, population‐adjusted evidence of an intensifying coastal per‐capita risk shift in 2025. Therefore, this study aims to provide a comprehensive mapping of the evolving spatiotemporal dynamics. By identifying dynamic and population‐adjusted hotspots and clustering patterns, these insights offer valuable information for designing and implementing targeted public health interventions and adaptive control strategies [6].

## 2. Methods

### 2.1. Study Design and Setting

We conducted a retrospective spatiotemporal ecological analysis of dengue fever incidence across all 64 administrative districts of Bangladesh from 1 January 2021 to 31 December 2025. This study is reported in accordance with the STROBE guidelines for observational studies (Table S12). This ecological surveillance approach is well‐suited for identifying high‐risk clusters in resource‐limited settings, where detailed individual‐level or covariate data may not be routinely available (as in many low‐ and middle‐income countries [LMICs], dengue surveillance systems) Two complementary spatial analytic frameworks were applied:

1. Global and local spatial autocorrelation using Moran’s I and *Getis-Ord Gi∗* to identify static clustering and population‐adjusted risk hotspots, particularly in 2025.
2. Space–time scan statistics using SaTScan [11, 12] to detect dynamic high‐ and low‐rate clusters over the full study period.

All analyses were population‐adjusted to account for underlying risk heterogeneity.

### 2.2. Dengue Case Definition

Dengue cases included in this analysis were suspected and laboratory‐confirmed dengue admissions reported by the Directorate General of Health Services (DGHS). A suspected dengue case was defined as an acute febrile illness with two or more of the following manifestations: headache, retro‐orbital pain, myalgia, arthralgia, rash, or bleeding manifestations, consistent with national and WHO clinical criteria. Laboratory confirmation (where performed) was based on NS1 antigen detection, IgM/IgG serology, or PCR. The aggregate district‐level counts from DGHS daily/weekly press releases represent hospital‐reported suspected and confirmed dengue admissions. No individual‐level diagnostic details were available in the public aggregate data.

### 2.3. Data Sources

We collected yearly aggregated district‐level dengue case admission counts (2021–October 2025) from daily press releases published by the DGHS [13]. All 64 districts were included in the final dataset, with no missing data across the study period. However, we had to employ annualization, as only partial‐year data of 2025, up to 6th November 2025 (310 days), were available during starting of this study. These data were annualized assuming uniform seasonality using this formula: *C*_2025,annual_ = *C*_Jan–Oct_ × 365/310. We used Population and Housing Census 2022 Preliminary Report, compiled and published by Bangladesh Bureau of Statistics (BBS) as the primary source of population data [14]. We applied a uniform 1.12% annual national growth rate on population data of 2022 [14]. For example, populations for 2025 were projected using a constant annual growth rate of 1.12%, applied over 3 years: POP_2025_ = POP_2022_ × (1 + 0.0112)^3^. Over the study period, the average national population (2021–2025) was 167,003,180. Population data used in the study are also incorporated in Table S6. Bangladesh has 64 districts, boundary of which (longitude and latitude in decimal degrees) and other relevant information for conducting spatial analysis were provided by Bangladesh Subnational Administrative Boundaries (ADM0–ADM4), in the format of shapefiles (.shp) and associated geospatial data, which are available from the United Nations Office for the Coordination of Humanitarian Affairs (OCHA) through the Humanitarian Data Exchange (HDX) portal at https://data.humdata.org/dataset/cod-ab-bgd [15]. The dataset is released under the Creative Commons Attribution 3.0 Intergovernmental Organization (CC BY 3.0 IGO) license [15]. Centroids for each district were calculated and saved using GeoDa software [16]. In production of maps and plots, EPSG:3857 (Web Mercator) was used as the projection for visualization consistency. Geographic data used in the study are also incorporated in Table S7.

### 2.4. Inclusion/Exclusion Criteria

All reported dengue cases from all 64 districts during the study period (1 January 2021 to 6 November 2025, annualized) were included. No districts or time periods were excluded due to unreliable data.

### 2.5. Age and Sex Aggregation

Age and sex breakdowns were not available in aggregate DGHS press releases.

### 2.6. Data Cleaning and Validation

All district‐level case and population data were cross‐checked for consistency across DGHS weekly bulletins and press releases. The absence of any missing values was confirmed via DGHS archival reports [13]. Data entry errors were carefully avoided.

### 2.7. Spatial Statistical Analyses

All spatial statistical analyses were conducted using Python 3.14.0 [17] within a Windows 10 (64 bit) environment. Data manipulation and management were performed using Pandas v2.3.3 and NumPy [18], while geospatial operations relied on GeoPandas v1.1.1 [19], libpysal v4.13.0, and the esda module [20]. Visualization of spatial results was accomplished using Matplotlib v3.10.7 [21] and contextily v1.6.2 for basemap integration [22], with python‐docx v1.2.0 used for reporting. Additional statistical analyses were supported by the statsmodels library [23]. All code was written and edited using Sublime Text software (Sublime HQ Pty Ltd., Woollahra, Sydney) and compiled with the respective compilers. Study definitions and geographical boundaries are detailed in Table S2.

For global and local spatial autocorrelation analyses, spatial weights were constructed using a Queen contiguity matrix (shared boundary or vertex), which was row‐standardized. Global spatial autocorrelation was assessed using Moran’s I, computed separately for raw case counts from 2021 to 2025 and for the 2025 annualized incidence rate per 100,000 population. Local hotspot analysis was performed using the Getis‐Ord *Gi∗* statistic to identify statistically significant hot and cold spots. Hotspots were defined as districts with *Gi∗* > 0 and *p* < 0.05, while cold spots were defined as districts with *Gi∗* < 0 and *p* < 0.05. Overlap analysis was conducted to identify districts that were significant in both raw 2025 cases and rate‐adjusted 2025 incidence.

Space–time cluster detection was performed using SaTScan v10.3.3 [11, 12] with a prospective space–time discrete Poisson model. The prospective approach was chosen over a purely retrospective model because it simulates an ongoing surveillance system and detects clusters as they emerge, which is directly relevant for real‐time public health monitoring, while our complete retrospective dataset allows validation of the method’s performance. The spatial window was specified as circular with a maximum size of 50% of the population to detect large urban clusters, and the temporal window was set to a maximum of 50% of the study period, aggregated in 2‐year units with a minimum duration of 1 year, to capture multiyear cycles and reduce climatic noise. Statistical significance was evaluated using 999 Monte Carlo replications with a threshold of *p* < 0.05. Detailed parameter specifications are provided in Table S3.

The scan statistic log‐likelihood ratio (LLR) was calculated using the formula, LLR = cln(*c*/*e*) + (*C* − *c*)ln((*C* − *c*)/(*C* − *e*)), where, *c* = observed cases in window, *e* = expected cases, and *C* = total cases. Relative risk (RR) was computed as RR = *c*/*e*/(*C* − *c*)/(*C* − *e*), where, *c* = observed cases in window, *e* = expected cases, and *C* = total cases. Annualized district‐level incidence for 2025 was calculated as Rate_2025_ = (*C*_2025,annual_ × 100000)/Pop_2025_. The national average incidence (2021–2025) was 76.4 per 100,000/year.

### 2.8. Temporal Standardization

Population projections for 2025 were calculated using the method described in the Population Data Source section above. Dengue case counts for 2025 were reported for 310 days (January 1 to November 6, 2025). To enable comparison with full‐year data from 2021–2024, the 2025 case counts were annualized to a 365‐day equivalent using the formula described in the Dengue Case Data section and expressed as incidence rates per 100,000 population. This adjustment accounts for the incomplete reporting period in 2025 while standardizing incidence relative to projected mid‐year population estimates. Annualized incidence rates were used in all 2025 spatial analyses.

### 2.9. Sensitivity Analysis for Annualization

To evaluate the robustness of the uniform annualization method for the partial 2025 data (310 days), we compared the annualized estimates with the actual complete‐year 2025 case counts obtained from subsequent DGHS press releases. The uniform annualization method showed moderate agreement overall (mean absolute percentage difference ≈48%), with better performance in high‐burden districts such as Dhaka (17% difference). A detailed comparison is presented in Table S14.

### 2.10. Visualization and Map Production

All choropleth and cluster maps were generated in Python using GeoPandas [19] and Matplotlib [21] with a uniform Web Mercator projection (EPSG:3857). Contextual basemaps were obtained from *OpenStreetMap* [24] tiles via the *contextily* library [22]. District labels were placed using centroid coordinates calculated from shape files available from the HDX [15] and within the *GeoDa Software environment* [16]. All figures were verified for scale and alignment consistency before export.

### 2.11. Computational Environment

All analyses were conducted on Windows 10 (64 bit) using Python 3.14.0 [17] and SaTScan v10.3.3 [11]. Random seeds were fixed manually at 1234567 in the SaTScan parameter/session (.prm) file to ensure reproducibility of Monte Carlo simulations.

### 2.12. Statistical Significance

All spatial and space–time clusters were evaluated at an alpha level of 0.05. Monte Carlo *p* values were derived from 999 replications, with clusters considered significant if *p* < 0.05.

## 3. Ethical Approval

This study used publicly available, aggregated surveillance data with no individual identifiers; therefore, no ethical approval was required. All epidemiological and population data were obtained from press releases made public by the DGHS [13] and published census report by BBS [14] and were analyzed in compliance with the principles of the Declaration of Helsinki.

## 4. Results

### 4.1. Data Summary

The analysis of dengue incidence covered a five‐year study period, from 1 January 2021 to 31 December 2025, involving an estimated total of 637,626 dengue cases across 64 districts (*Gi∗*) for the initial overview, with the subsequent spatiotemporal analysis in SaTScan [11]. The case count for the final year (2025) was determined by annualizing data available up to November 6th, 2025, using estimation methods detailed in the relevant section of this article. Based on an average population of 167,003,180 [11, 14], the calculated national incidence rate for this period was 76.4 cases per 100,000 people per year. For detailed sensitivity analysis of *Getis-Ord Gi*∗, see Supporting Figures 1–3. Sensitivity analyses confirmed the robustness of the primary findings.

## 5. Global and Local Spatial Autocorrelation (*Gi∗* and Moran’s I)

Moran’s I measures (Table 1) whether districts with similar dengue levels are geographically clustered. For total case counts (raw numbers), there was no significant clustering—high‐case districts were not consistently next to each other. However, when adjusted for population size (incidence rate), 2025 showed strong and significant clustering (*p* = 0.001), meaning there are districts with high risk per person.

**TABLE 1:** Global Moran’s I for dengue incidence, 2021–2025.

Year	Variable	Moran’s I	*p* value	*z* value
2021	Raw cases	−0.0030	0.104	1.17
2022	Raw cases	−0.0251	0.490	−0.29
2023	Raw cases	0.0036	0.161	0.46
2024	Raw cases	0.0284	0.063	1.75
2025	Raw cases	0.0462	0.087	1.60
2025	Rate per 100 k	0.3281	0.001	4.55

**FIGURE 1:**
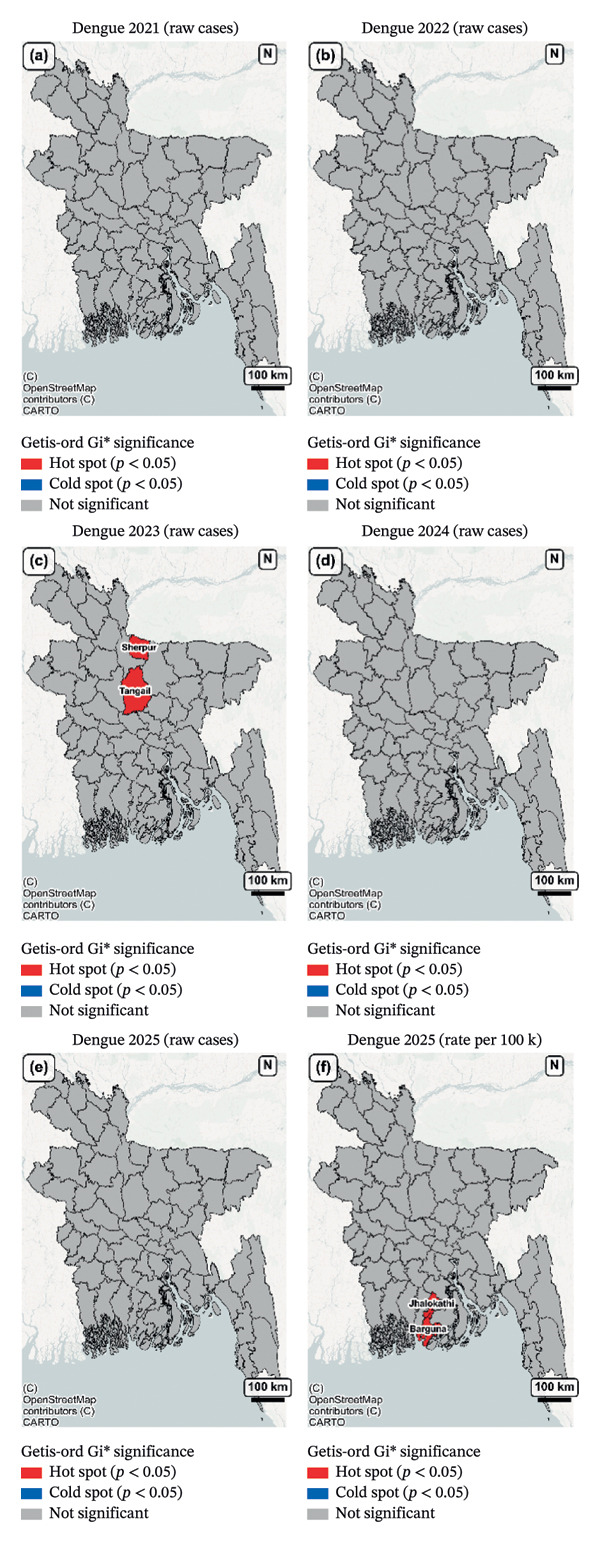
Spatial evolution of dengue hot spots (Getis‐Ord Gi∗) in Bangladesh, 2021–2025. (a) 2021 (raw cases): shows district‐level spatial clustering for raw cases; no statistically significant hot spots or cold spots are detected across the country (all districts remain grey/non‐significant). (b) 2022 (raw cases): displays spatial clustering for raw cases; similar to 2021, all districts show no statistically significant hot spots or cold spots. (c) 2023 (raw cases): identifies a prominent central hot spot (*p* < 0.05) highlighted in red across the Sherpur and Tangail districts. (d) 2024 (raw cases): displays district‐level spatial significance for raw cases, showing non‐significant patterns across the map. (e) 2025 (raw cases): displays raw case clustering across districts, remaining non‐significant overall. (f) 2025 (rate per 100k/rate‐adjusted incidence): highlights statistically significant southern coastal hot spots (*p* < 0.05) in red across Jhalokathi and Barguna districts when adjusting case counts per 100,000 population. Legend: red = hot spot (*p* < 0.05), light blue = cold spot (*p* < 0.05), grey = not significant.

These maps (Figure 1) show where dengue was unusually high compared to neighboring districts. In 2021‐2022, no clear hotspots existed. By 2023, central districts such as Tangail and Sherpur had significantly more total cases. In 2025, Greater Dhaka remained a hotspot for total case volume, but when adjusted for population, the highest risk per person shifted to southern coastal districts (Jhalokathi and Barguna). No district was a hotspot in both metrics at the same time.

### 5.1. Significant *Gi∗* Hotspots: Raw Cases (2023) and Rate‐Adjusted Incidence (2025)

**FIGURE 2:**
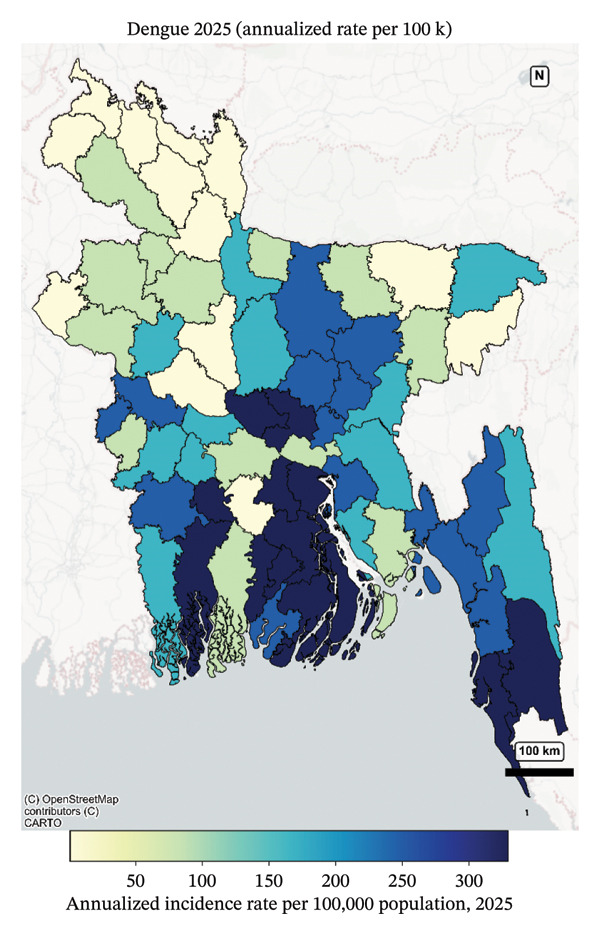
Annualized dengue incidence rate per 100,000 in Bangladesh by district, 2025. The choropleth colors represent the annualized dengue incidence rate per 100,000 population. Rates range from low (light yellow/green, < 50 cases per 100,000) to high (dark purple/black, ≥ 300 cases per 100,000), showing the continuous spectrum of dengue burden across the country.

Two distinct sets of geographical hotspots for dengue incidence were identified using the *Gi∗* statistics. In 2023, two central districts, Tangail (*Gi∗* = 0.093994, *p* = 0.042) and Sherpur (*Gi∗* = 0.090792, *p* = 0.049), exhibited significantly higher raw case numbers than expected. These locations represented “true hotspots” based purely on the sheer volume of disease activity, even after considering neighboring district counts. Conversely, the 2025 analysis, which adjusted for population rate differences (Figure 2), identified two separate hotspots: Jhalokathi (*Gi∗* = 0.070577, *p* = 0.006) and Barguna (*Gi∗* = 0.061848, *p* = 0.019). These two districts demonstrated significantly elevated dengue incidence rates relative to their surrounding areas. Despite lower total cases, their smaller populations meant that each infection represented a larger public health burden.

Here, Figure 2 shows that the highest per capita rates (72.5–329.4 per 100,000) were found in southern and eastern coastal zones. Central metropolitan areas show a high absolute burden and moderate per capita risk. This map shows per‐capita burden in terms of number of cases per 100,000 persons, not total cases. Darker shades indicate higher incidence rates. Southern and eastern coastal districts had the highest risk per resident, while Dhaka had many cases but lower per‐person risk due to its massive population. For detailed sensitivity analysis, of *Getis-Ord Gi∗, see* Table S9 and Table S8.

## 6. Space–Time Cluster Detection (SaTScan)

**FIGURE 3:**
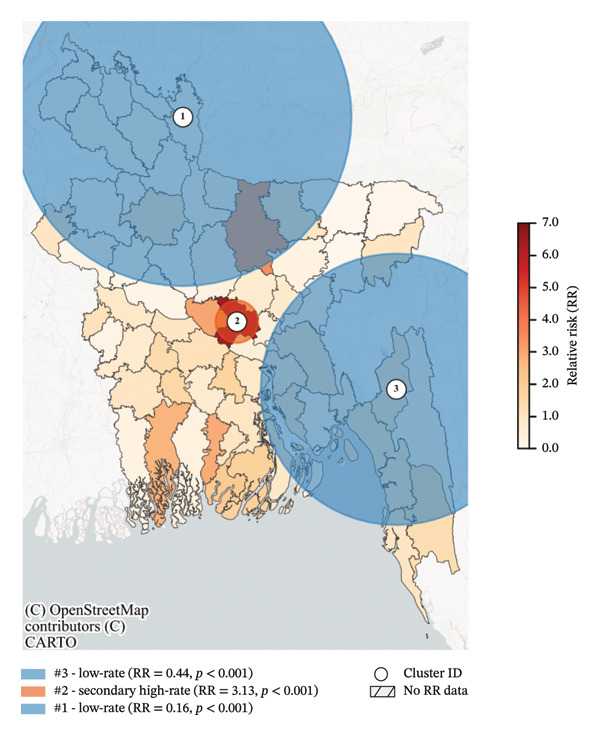
Space–time clusters of dengue incidence (50% spatial and 50% temporal base model, for a period between 1st January 2024 to 31st December 2025) in Bangladesh. These clusters are marked over this choropleth map illustrating individual dengue relative risk by district over the years of 2021–2025. The color scale is sequential, ranging from white (lowest risks, RR ∼ 0.0) through yellow, orange ,and red, indicating the highest risks (RR ∼ 7.0). Individual clusters are marked with numbers. Cluster 1 = Kurigram, Lalmonirhat, Rangpur, Gaibandha, Nilphamari, Sherpur, Joypurhat, Jamalpur, Dinajpur, Bogura, Thakurgaon, Panchagarh, Mymensingh, Naogaon, Netrokona, Sirajganj, Natore, Tangail, Kishoreganj, Rajshahi, Chapainawabganj, Sunamganj, Pabna. Cluster 2 = Dhaka, Manikganj. Cluster 3 = Khagrachhari, Rangamati, Feni, Chattogram, Cumilla, Noakhali, Bandarban, Lakshmipur, Brahmanbaria, Chandpur, Habiganj, Bhola, Narsingdi, Maulvibazar, Narayanganj, Munshiganj, Shariatpur.

In space–time cluster analysis (Table 2, more detailed description of the clusters in Table S4), all detected clusters were highly significant (*p* = 0.001) with recurrence intervals of 2000 years, indicating very strong spatiotemporal signals. The space–time scan analysis revealed distinct geographic variations in dengue transmission across Bangladesh during 2024‐2025. A significant high‐rate cluster was detected in the Dhaka–Manikganj region (Figure 3), where the number of reported dengue cases was nearly three times higher than expected based on the population size. The estimated RR (RR = 3.13) indicates that residents in this central urban cluster were more than three times as likely to contract dengue compared to those living outside the cluster. In contrast, two extensive low‐rate clusters were identified: one encompassing 23 northern districts and another covering 17 southeastern districts. The northern cluster exhibited approximately 82% fewer cases than expected (O/E = 0.18, RR = 0.16), while the southeastern cluster showed a 53% deficit in observed cases (O/E = 0.46, RR = 0.43). Together, these results indicate a strong concentration of dengue transmission in the central urban belt, particularly around Dhaka, while the northern and southeastern regions remained relatively less affected during the study period. For more details, please see Table S10 and Table S11.

**TABLE 2:** Statistically significant space–time clusters (*p* = 0.001), time‐period for all clusters: 1st January 2024 to 31st December 2025.

Rank	Type	Districts (*n*)	Center (longitude, latitude)	Radius (km)	Time frame	Population	Observed cases	Expected cases	O/E	Relative risk (RR)	Log‐likelihood ratio (LLR)	*p* value
1	Low rate	23	(89.6594°E, 25.812°N)	∼205	2024–2025	60,868,547	17,118	93,996.65	0.18	0.16	52,920.95	0.001
2	High rate	2 (Dhaka, Manikganj)	(90.2508°E, 23.787°N)	∼26	2024–2025	16,475,622	73,433	25,442.58	2.89	3.13	31,777.05	0.001
3	Low rate	17	(91.9915°E, 23.1103°N)	∼165	2024–2025	46,729,120	33,561	72,163.11	0.47	0.44	14,197.62	0.001

*Note:* Cluster 1 = Kurigram, Lalmonirhat, Rangpur, Gaibandha, Nilphamari, Sherpur, Joypurhat, Jamalpur, Dinajpur, Bogura, Thakurgaon, Panchagarh, Mymensingh, Naogaon, Netrokona, Sirajganj, Natore, Tangail, Kishoreganj, Rajshahi, Chapainawabganj, Sunamganj, Pabna. Cluster 2 = Dhaka, Manikganj. Cluster 3 = Khagrachhari, Rangamati, Feni, Chattogram, Cumilla, Noakhali, Bandarban, Lakshmipur, Brahmanbaria, Chandpur, Habiganj, Bhola, Narsingdi, Maulvibazar, Narayanganj, Munshiganj, Shariatpur.

### 6.1. District‐Level Risk Estimates (2021–2025) Space–Time Clusters (SaTScan, 2024–2025; see Figure 3, Table S5 for More Details)

• Cluster 1— Northern low‐rate (23 districts): Kurigram, Lalmonirhat, Rangpur, Gaibandha, Nilphamari, Sherpur, Jamalpur, Joypurhat, Bogura, Dinajpur, Panchagarh, Thakurgaon, Mymensingh, Naogaon, Netrokona, Sirajganj, Natore, Tangail, Rajshahi, Kishoreganj, Chapainawabganj, Pabna, and Sunamganj. In this cluster, 82% fewer cases than expected (O/E = 0.18, RR = 0.16); sustained low‐transmission zone.
• Cluster 2—Dhaka Urban High‐Rate (2 districts): Dhaka, Manikganj. In this cluster, nearly 3 times more than expected cases (O/E = 2.89, RR = 3.13) were found; 11.5% of national cases from 9.9% of population.
• Cluster 3—Southeastern Low‐Rate (17 districts): Khagrachhari, Rangamati, Feni, Chattogram, Cumilla, Noakhali, Lakshmipur, Brahmanbaria, Bandarban, Chandpur, Habiganj, Maulvibazar, Bhola, Narsingdi, Narayanganj, Munshiganj, and Shariatpur. In this cluster, 53% fewer cases than expected (O/E = 0.47, RR = 0.44), including hill tracts and coastal plains.

District‐level dengue risk analysis (2021–2025), comparing observed to population‐expected cases, revealed stark regional contrasts. Dhaka ranked highest with 245,266 observed cases vs. 56,884.98 expected, yielding an O/E ratio of 4.31 and RR = 6.38, indicating over four times the expected burden. Mymensingh followed (O/E 3.16, RR 3.43) and Manikganj (O/E 2.97, RR 3.02). In contrast, northern districts showed extremely low activity: Sunamganj had only 1% of expected cases; Kurigram (O/E 0.060, RR 0.060) and Panchagarh (O/E 0.064, RR 0.063) confirmed minimal risk. Full district‐level estimates are in Table S5.

A sensitivity analysis comparing the annualized 2025 estimates with actual full‐year case counts subsequently released by DGHS revealed that although some smaller districts exhibited larger deviations, the key spatial patterns remained consistent. Jhalokathi and Barguna continued to rank among the districts with the highest per‐capita incidence, and Barguna recorded the second‐highest total burden in 2025 with 9534 cases. Detailed comparison is presented in Table S14.

## 7. Discussion

Our five‐year (2021–2025) spatiotemporal ecological analysis identifies a dual dengue risk geography in Bangladesh, marked by persistent hyperendemicity in the central urban core and emerging high‐risk zones in southern coastal districts. This pattern reflects the entrenched urban burden alongside geographic expansion, consistent with earlier reports of evolving dengue transmission [6, 8, 25] but provides a new, nuanced quantification of this shift.

The space–time scan revealed a highly significant high‐rate cluster (*p* = 0.001) encompassing Dhaka and Manikganj districts during 2024‐2025. This cluster, with 16.48 million residents and a radius of ∼26 km, reported 73,433 observed cases versus 25,443 expected (O/E = 2.89; RR = 3.13). Dhaka district alone exhibited 245,266 observed cases versus 56,885 expected (O/E = 4.31; RR = 6.38), confirming that residents were over six times more likely to experience dengue than those outside the district. This finding aligns with the historical pattern identified by Banu et al. (2011), who found Dhaka to be the persistent “most likely cluster” with a peak RR of 233.3 in 2004‐2005. While the absolute RR in our study is lower, it confirms Dhaka’s unwavering role as the primary driver of the national case burden over 2 decades. This highlights Dhaka as the national epicenter, driving the absolute case burden [6]. The stark contrast between the magnitude of RR in our study and the study by Banu et al. probably reflects the rising ubiquity of dengue infection in Bangladesh, resulting in increased risk in all areas and districts, drawing the ratio of risks between high‐risk cluster and every other area lower [5, 25].

However, a critical evolution is evident. Historically, Dhaka was the dominant focus, while secondary clusters previously appeared transiently in coastal cities such as Khulna and Chittagong [25]. Whereas early studies suggested contraction of clusters in the 2000 s, our analysis demonstrates pronounced geographic expansion across all 64 districts. Local hotspot analysis using *Getis-Ord Gi∗* demonstrates a geographic dissociation: While raw case hotspots remain concentrated in Greater Dhaka, population‐adjusted hotspots have shifted to southern coastal districts, particularly Jhalokathi and Barguna in 2025. This reveals a fundamental shift in the risk landscape. Banu et al. identified secondary clusters in coastal cities such as Khulna (RR = 21.64 in 2000) and Chittagong, but noted these were transient [25]. In contrast, our study, along with Hossain et al., shows that high risk in the South has become a persistent feature. Hossain et al. identified a stable hotspot in the southern region from 2019–2023, with Bagerhat, Jhalokathi, and Patuakhali emerging as new hotspots in 2022‐2023. Our 2025 data confirm and sharpen this focus, statistically pinpointing Jhalokathi and Barguna as the current epicenters of per‐capita risk [6]. The consistency of these patterns across both annualized and actual full‐year 2025 data further supports the robustness of our primary spatiotemporal findings (Table S14).

Dhaka’s share of national cases declined from 51% in 2019 to 34% in 2023, while per‐capita risk in these coastal districts has increased, emphasizing the distinction between absolute burden and individual‐level risk [6]. The later availability of actual full‐year 2025 data allowed us to validate the uniform annualization approach used in the primary analysis. While some district‐level differences were observed, the core conclusions regarding Greater Dhaka as the high‐volume urban hyperendemic area and the southern coastal districts (particularly Jhalokathi and Barguna) as emerging high‐burden areas remained robust.

While our purely descriptive spatial analysis identifies where transmission risk has shifted, it does not formally test environmental or socioeconomic drivers. The following interpretations are therefore hypothesis‐generating, based on the existing literature rather than direct statistical evidence from this study. Environmental factors strongly shape these patterns. In Dhaka, UHIs with land surface temperatures of 27°C–32°C accelerate mosquito larval development and virus replication in *Aedes aegypti*, sustaining hyperendemic transmission [7]. *Aedes albopictus* also contributes to urban transmission, historically overlapping with high dengue incidence and complicating control measures targeting only *Aedes aegypti* [26]. In coastal districts, high groundwater salinity and arsenic contamination necessitate rainwater harvesting, often stored in uncovered containers, creating prolific breeding habitats that increase per‐capita dengue risk [25, 27]. This shift in the high‐risk paradigm from purely urban density to include rural, environmentally‐driven vulnerability is a critical insight for public health planning. Future studies incorporating ecological regression or covariate‐adjusted models (e.g., with salinity, rainfall, or land‐use data) are needed to rigorously test these associations.

Temporal trends in our study indicate a persistent high‐rate cluster during 2024‐2025, corroborating ARIMA and SARIMA model predictions of an astronomical case burden in a previous study [5]. Seasonal peaks have shifted earlier, signaling temporal instability in transmission and highlighting the need for proactive, adaptive public health responses [6]. Comparable patterns are observed globally: in São Paulo, Brazil, over 93% of dengue cases occurred in UHIs, while in temperate Europe, climate change is facilitating *Aedes* expansion, emphasizing the importance of climate‐aware vector control strategies [28, 29].

Methodologically, combining *Getis-Ord Gi∗* and SaTScan was crucial to reveal this dual geography. *Gi∗* identified high case volume in Dhaka versus rising per‐capita risk in coastal districts, while SaTScan pinpointed the 2024‐2025 high‐rate cluster and its timing. Banu et al. relied solely on SaTScan, which is optimal for finding compact, high‐case clusters like Dhaka. Hossain et al. effectively used *Gi∗* and local Moran’s I to reveal the widespread southern hotspots and northern coldspots. Our study synthesizes these approaches: SaTScan captured the dynamic Dhaka–Manikganj cluster, while *Gi∗* with population‐adjustment exposed the high‐rate coastal districts—a nuance that might be diluted in a purely case‐based SaTScan analysis or a raw‐case *Gi∗* map. Sensitivity analyses adjusting spatial weights, population data, and SaTScan parameters consistently confirmed both urban clusters and coastal hotspots, demonstrating robustness. Using either method alone would have obscured either coastal risk or outbreak dynamics.

The marked spatial heterogeneity observed in our analysis, with dengue transmission highly concentrated in the central urban belt, underscores the need for geographically targeted vector control and surveillance strategies, with priority given to high‐risk urban centers. Such targeted approaches are essential given the limited resources available and the expanding geographic scope of dengue risk. In hyperendemic urban centers like Dhaka, sustained intensive vector control and case management remain essential. In emerging coastal hotspots such as Jhalokathi and Barguna, proactive early‐warning surveillance, community environmental management tailored to local water storage practices, and enhanced mosquito monitoring are urgently required. Strategic resource allocation must address both entrenched urban epidemics and rising coastal threats.

### 7.1. Strengths

• Comprehensive nationwide analysis of all 64 districts over five years
• Integration of complementary spatial methods (*Gi∗*, SaTScan) revealing distinct risk patterns
• Population‐adjusted analyses differentiating between absolute burden and per‐capita risk
• Use of prospective space–time scanning simulating real‐world surveillance
• Complete geographic coverage with no missing data

### 7.2. Limitations

This study has several limitations. As an ecological analysis, the findings are subject to the ecological fallacy and cannot be used to infer causation or individual‐level risk factors. Potential surveillance bias may exist, with possible underdetection in remote rural areas and relatively better reporting in districts with greater healthcare access, including some coastal regions. We relied on projected population data and annualized 2025 case counts. No multivariate regression was performed to adjust for potential confounders such as rainfall, socioeconomic status, or vector density. These limitations should be considered when interpreting the results. Future studies incorporating individual‐level or covariate‐adjusted spatial regression models are recommended.

## 8. Conclusion

In conclusion, this integrated spatiotemporal analysis corroborates the historical shift of dengue risk from a Dhaka‐centric problem to a national concern, identifying a clear dual risk pattern of urban hyperendemicity and emerging coastal per‐capita risk. These insights, when coupled with environmental analyses and future forecasts, underscore the urgent need for adaptable, targeted interventions and resource allocation to combat the escalating and geographically diversifying dengue burden in Bangladesh.

**Nomenclature**
ADM: Administrative division
ARIMA: Autoregressive integrated moving average
BBS: Bangladesh Bureau of Statistics
DGHS: Directorate General of Health Services
EPSG: European Petroleum Survey Group
*Gi∗*
: *Getis-Ord Gi∗* statistic
HDX: Humanitarian data exchange
KNN: K‐nearest neighbors
LLR: Log‐likelihood ratio
LST: Land surface temperature
O/E: Observed‐to‐expected ratio
OCHA: United Nations Office for the Coordination of Humanitarian Affairs
RR: Relative risk
SARIMA: Seasonal autoregressive integrated moving average
UECs: Urban environmental components
UHI: Urban heat island.

## Author Contributions

Pratyay Hasan: conceptualization, methodology, software, data curation, formal analysis, supervision, visualization, project administration, resources, writing–original draft, and writing–review and editing.

Tazdin Delwar Khan: conceptualization, methodology, data curation, formal analysis, supervision, visualization, project administration, resources, writing–original draft, and writing–review and editing.

Mohammad Saiful Islam: conceptualization, methodology, data curation, formal analysis, supervision, visualization, project administration, resources, writing–original draft, and writing–review and editing.

Mohd. Arifuzzaman: conceptualization, methodology, software, data curation, formal analysis, supervision, visualization, project administration, resources, and writing–review and editing.

Mohammad Emdadul Haque: conceptualization, methodology, software, data curation, formal analysis, supervision, visualization, project administration, resources, writing–original draft, and writing–review and editing.

## Funding

This research did not receive any specific grant from funding agencies in the public, commercial, or not‐for‐profit sectors.

## Disclosure

The lead author (Pratyay Hasan) affirms that this manuscript is an honest, accurate, and transparent account of the study being reported; that no important aspects of the study have been omitted; and that any discrepancies from the study as planned (and, if relevant, registered) have been explained.

The authors confirm that they have adhered to relevant EQUATOR guidelines, and the reporting method is referenced in the abstract and methods section of this paper.

All authors have read and approved the final version of the manuscript. Pratyay Hasan had full access to all of the data in this study and takes complete responsibility for the integrity of the data and the accuracy of the data analysis. The authors are solely responsible for the analyses, interpretations, and conclusions presented in this manuscript.

## Consent

Patients and/or the public were not involved in the design, conduct, analysis, or dissemination of this research due to its ecological nature, relying solely on aggregated, publicly available surveillance data from the Directorate General of Health Services (DGHS) and Bangladesh Bureau of Statistics (BBS). No individual patient data or direct human subjects were accessed, making direct PPI inappropriate. Future studies on dengue interventions will prioritize PPI to ensure community relevance. The corresponding author takes responsibility for ethical considerations.

## Conflicts of Interest

The authors declare no conflicts of interest.

## Supporting Information

Additional supporting information can be found online in the Supporting Information section.

## Supporting information


**Supporting Information** Supporting Figures: (1) Supporting Figure 1: Sensitivity analysis for different scenarios (TIF). (2) Supporting Figure 2: Sensitivity analysis for different spatial weights (TIF). (3) Supporting Figure 3: Sensitivity analysis for satscan combination parameters (TIF). Supporting Documents: (1) Supporting Table S1‐S11: Comprehensive study materials and sensitivity analysis (DOCX). (a) Table S1: Study overview and case Definition 1. (b) Table S3: Full setup of software (SaTScan) 2. (c) Table S4: Detailed description of Clusters 2. (d) Table S5: District level risk 3. (e) Table S6: Population data used in Study 4. (f) Table S7: Full geographic Data 12. (g) Table S7: Full geographic data used in Study 13. (h) Table S8: Gi∗ Values 15. (i) Table S9: Gi∗ Sensitivity Summary 19. (j) Table S10: (SaTScan) Sensitivity analysis of high‐risk Clusters 24. (k) Table S11: (SaTScan) Sensitivity analysis of low‐risk Clusters 25. (2) Supporting Table S12: STROBE Statement (DOCX)—Completed checklist for Strengthening the Reporting of Observational Studies in Epidemiology. (3) Supporting Table S13: Districts dengue cases 2021_2025.xlsx—The complete dengue data for 64 districts of Bangladesh. (4) Supporting Table S14: All 2025 Data—Comparison of partial, annualized, and actual full‐year 2025 dengue cases by district.

## Data Availability

The data that support the findings of this study are available in the Supporting information of this article.
