# Supplementary material for: Urban Hyperendemicity and Emerging Coastal Per‐Capita Risk of Dengue in Bangladesh, 2021–2025: A Nationwide Spatiotemporal Ecological Analysis
Source: J Trop Med. 2026 Aug 7;2026:3012361. doi: 10.1155/jotm/3012361 (PMC13451933; doi:10.1155/jotm/3012361)
Supplement: Supplementary file 1 [file JOTM-2026-3012361-s001.zip › Supplementary Tab.S1-S11 All Materials and Sensitivity Analysis.docx]

# Supplementary Materials

[***Tab.S1*** Study overview and case definition	1](#_Toc231595077)

[***Tab.S3*** Full setup of software (SaTScan)	2](#_Toc231595078)

[***Tab.S4*** Detailed description of clusters	2](#_Toc231595079)

[***Tab.S5*** District level risk	3](#_Toc231595080)

[***Tab.S6*** Population data used in the study	4](#_Toc231595081)

[***Tab.S7*** Full geographic data used in this study	12](#_Toc231595082)

[***Tab.S8*** Gi* Values	15](#_Toc231595083)

[***Tab.S9*** Gi* Sensitivity summary	19](#_Toc231595084)

[***Tab.S10*** (SaTScan) Sensitivity Analysis of High-Risk Clusters	24](#_Toc231595085)

[***Tab.S11*** (SaTScan) Sensitivity Analysis of Low-Risk Clusters	25](#_Toc231595086)

# Study Overview

***Tab.S1*** Study overview and case definition

| **Component** | **Required Information** | **Location** |
| --- | --- | --- |
| **Study Period** | Exact start and end dates of case collection: **1 January 2021 – 31 December 2025**. | Abstract/Methods |
| **Study Population** | Average population figure used for rate calculation: **167,003,180** [16, 19, 32]. | Methods/Results |
| **Case Data** | Total number of cases reported: **637,626**. | Abstract/Results |
| **Annualization** | State the exact cutoff date (**6 November 2025**) and methods used to annualize the final year's data to **31 December 2025**. | Methods |

## 2. Geographical Data and Boundaries

***Tab.S2*** Geographic data and boundaries

| **Component** | **Required Information** | **Location** |
| --- | --- | --- |
| **Geographic Units** | The specific administrative level analyzed (e.g., district or *upazila*). | Methods |
| **Input Files** | State the source and type of geographic files used (e.g., district-level shapefile named "**64 districts.shp**"). | Methods/Supplementary |

## 3. SaTScan Cluster Analysis

The SaTScan analysis details are critical for reproducibility and must be transparently reported.

## A. SaTScan Parameter Settings (Supplementary Appendix S1.1)

***Tab.S3*** Full setup of software (SaTScan)

| **Parameter** | **Setting** |
| --- | --- |
| **Software Version** | SaTScan v10.3.3 |
| **Type of Analysis** | **Prospective Space-Time** |
| **Probability Model** | **Discrete Poisson** |
| **Scan Objective** | High or Low Rates |
| **Time Aggregation** | 2 Years (Time frame is 2024/1/1 to 2025/12/31) |
| **Max Spatial Cluster Size** | 50% of population at risk |
| **Max Temporal Cluster Size** | 50% of study period |
| **Inference** | 999 Monte Carlo Replications, random seed 1234567, manually set at parameter (.prm) file. |

## B. Detailed Cluster Results

A table summarizing all significant clusters, including their exact composition and statistical metrics (see previous response for suggested table format).

***Tab.S4*** Detailed description of clusters

| **Metric** | **High-Rate Cluster (Cluster 2)** | **Low-Rate Cluster (Cluster 1)** | **Low-Rate Cluster (Cluster 3)** |
| --- | --- | --- | --- |
| **Location IDs** | Dhaka, Manikganj | 23 Northern/Central Districts | 17 Eastern/Coastal Districts |
| **Center / Radius** | 23.7870° N, 90.2508° E / 25.81 km | 25.8120° N, 89.6594° E / 205.12 km | 23.1103° N, 91.9915° E / 164.73 km |
| **Time Frame** | 2024/1/1 to 2025/12/31 | 2024/1/1 to 2025/12/31 | 2024/1/1 to 2025/12/31 |
| **Relative Risk (RR)** | **3.13** | **0.16** | **0.44** |
| **P-value** | **0.001** | **0.001** | **0.001** |

## C. District-Level Risk Data

***Tab.S5*** District level risk

| District | Observed | Expected | O/E | RR |
| --- | --- | --- | --- | --- |
| Bagerhat | 2237 | 6227.58 | 0.36 | 0.36 |
| Bandarban | 2453 | 1857.4 | 1.32 | 1.32 |
| Barguna | 8252 | 3901.3 | 2.12 | 2.13 |
| Barishal | 12640 | 9923.6 | 1.27 | 1.28 |
| Bhola | 6643 | 7460.82 | 0.89 | 0.89 |
| Bogura | 2104 | 14416.89 | 0.15 | 0.14 |
| Brahmanbaria | 2560 | 12765.55 | 0.2 | 0.2 |
| Chandpur | 8533 | 10175.75 | 0.84 | 0.84 |
| Chapainawabganj | 365 | 7086.37 | 0.052 | 0.051 |
| Chattogram | 27827 | 35400.09 | 0.79 | 0.78 |
| Chuadanga | 1136 | 4764.34 | 0.24 | 0.24 |
| CoxsBazar | 13606 | 10899.73 | 1.25 | 1.25 |
| Cumilla | 9828 | 23983.32 | 0.41 | 0.4 |
| Dhaka | 245266 | 56883.12 | 4.31 | 6.38 |
| Dinajpur | 1812 | 12799.01 | 0.14 | 0.14 |
| Faridpur | 10010 | 8350.17 | 1.2 | 1.2 |
| Feni | 3396 | 6365.82 | 0.53 | 0.53 |
| Gaibandha | 495 | 9891.89 | 0.05 | 0.049 |
| Gazipur | 14965 | 20320.46 | 0.74 | 0.73 |
| Gopalganj | 3597 | 4999.79 | 0.72 | 0.72 |
| Habiganj | 370 | 9106.86 | 0.041 | 0.04 |
| Jamalpur | 2239 | 9650.67 | 0.23 | 0.23 |
| Jashore | 8556 | 11878.73 | 0.72 | 0.72 |
| Jhalokati | 3243 | 2552.53 | 1.27 | 1.27 |
| Jhenaidah | 5591 | 7743.94 | 0.72 | 0.72 |
| Joypurhat | 369 | 3692.46 | 0.1 | 0.099 |
| Khagrachhari | 1505 | 2756.99 | 0.55 | 0.54 |
| Khulna | 26610 | 10089.41 | 2.64 | 2.71 |
| Kishoreganj | 4108 | 12615.2 | 0.33 | 0.32 |
| Kurigram | 544 | 8992.14 | 0.06 | 0.06 |
| Kushtia | 7449 | 8299.26 | 0.9 | 0.9 |
| Lakshmipur | 8252 | 7486.24 | 1.1 | 1.1 |
| Lalmonirhat | 427 | 5514.62 | 0.077 | 0.077 |
| Madaripur | 8438 | 4991.95 | 1.69 | 1.7 |
| Magura | 5779 | 3988.53 | 1.45 | 1.45 |
| Manikganj | 17849 | 6015 | 2.97 | 3.02 |
| Maulvibazar | 160 | 8197.88 | 0.02 | 0.019 |
| Meherpur | 3103 | 2723.14 | 1.14 | 1.14 |
| Munshiganj | 3216 | 6275.21 | 0.51 | 0.51 |
| Mymensingh | 71912 | 22774.22 | 3.16 | 3.43 |
| Naogaon | 627 | 10750.4 | 0.058 | 0.057 |
| Narail | 4750 | 3044.83 | 1.56 | 1.56 |
| Narayanganj | 7160 | 15091.8 | 0.47 | 0.47 |
| Narsingdi | 7877 | 9977.7 | 0.79 | 0.79 |
| Natore | 1115 | 7180.52 | 0.16 | 0.15 |
| Netrokona | 1400 | 8975.45 | 0.16 | 0.15 |
| Nilphamari | 345 | 8078.69 | 0.043 | 0.042 |
| Noakhali | 2831 | 13995.92 | 0.2 | 0.2 |
| Pabna | 1862 | 11233.12 | 0.17 | 0.16 |
| Panchagarh | 290 | 4554.98 | 0.064 | 0.063 |
| Patuakhali | 12051 | 6668.38 | 1.81 | 1.82 |
| Pirojpur | 13143 | 4625.82 | 2.84 | 2.88 |
| Rajbari | 4702 | 4593.53 | 1.02 | 1.02 |
| Rajshahi | 2314 | 11253.89 | 0.21 | 0.2 |
| Rangamati | 780 | 2500.11 | 0.31 | 0.31 |
| Rangpur | 1603 | 12236.83 | 0.13 | 0.13 |
| Satkhira | 2680 | 8480.24 | 0.32 | 0.31 |
| Shariatpur | 4480 | 4997.86 | 0.9 | 0.9 |
| Sherpur | 1847 | 5798.17 | 0.32 | 0.32 |
| Sirajganj | 994 | 12963.06 | 0.077 | 0.075 |
| Sunamganj | 102 | 10406.4 | 0.0098 | 0.0096 |
| Sylhet | 1475 | 14890.62 | 0.099 | 0.097 |
| Tangail | 5462 | 15587.82 | 0.35 | 0.34 |
| Thakurgaon | 291 | 5921.86 | 0.049 | 0.049 |
| ``` |  |  |  |  |

# Population Data used in Cluster Analysis

***Tab.S6*** Population data used in the study

| District | Population | Year |
| --- | --- | --- |
| Bagerhat | 1595015 | 2021 |
| Bagerhat | 1613079 | 2022 |
| Bagerhat | 1631140 | 2023 |
| Bagerhat | 1649416 | 2024 |
| Bagerhat | 1667887 | 2025 |
| Bandarban | 475917 | 2021 |
| Bandarban | 481109 | 2022 |
| Bandarban | 486496 | 2023 |
| Bandarban | 491945 | 2024 |
| Bandarban | 497455 | 2025 |
| Barguna | 999369 | 2021 |
| Barguna | 1010530 | 2022 |
| Barguna | 1021838 | 2023 |
| Barguna | 1033277 | 2024 |
| Barguna | 1044865 | 2025 |
| Barishal | 2540055 | 2021 |
| Barishal | 2570450 | 2022 |
| Barishal | 2599219 | 2023 |
| Barishal | 2628306 | 2024 |
| Barishal | 2657788 | 2025 |
| Bhola | 1911027 | 2021 |
| Bhola | 1932514 | 2022 |
| Bhola | 1954150 | 2023 |
| Bhola | 1976052 | 2024 |
| Bhola | 1998176 | 2025 |
| Bogura | 3691458 | 2021 |
| Bogura | 3734300 | 2022 |
| Bogura | 3776116 | 2023 |
| Bogura | 3818392 | 2024 |
| Bogura | 3861183 | 2025 |
| Brahmanbaria | 3266299 | 2021 |
| Brahmanbaria | 3306559 | 2022 |
| Brahmanbaria | 3343590 | 2023 |
| Brahmanbaria | 3381035 | 2024 |
| Brahmanbaria | 3418908 | 2025 |
| Chandpur | 2603577 | 2021 |
| Chandpur | 2635748 | 2022 |
| Chandpur | 2665246 | 2023 |
| Chandpur | 2695111 | 2024 |
| Chandpur | 2725304 | 2025 |
| Chattogram | 9064406 | 2021 |
| Chattogram | 9169464 | 2022 |
| Chattogram | 9272112 | 2023 |
| Chattogram | 9375859 | 2024 |
| Chattogram | 9480021 | 2025 |
| Chuadanga | 1220012 | 2021 |
| Chuadanga | 1234066 | 2022 |
| Chuadanga | 1247895 | 2023 |
| Chuadanga | 1261863 | 2024 |
| Chuadanga | 1275996 | 2025 |
| CoxsBazar | 2792135 | 2021 |
| CoxsBazar | 2823265 | 2022 |
| CoxsBazar | 2854892 | 2023 |
| CoxsBazar | 2886866 | 2024 |
| CoxsBazar | 2919193 | 2025 |
| Cumilla | 6139625 | 2021 |
| Cumilla | 6212216 | 2022 |
| Cumilla | 6281800 | 2023 |
| Cumilla | 6352108 | 2024 |
| Cumilla | 6423292 | 2025 |
| Dhaka | 14558397 | 2021 |
| Dhaka | 14734025 | 2022 |
| Dhaka | 14898977 | 2023 |
| Dhaka | 15065801 | 2024 |
| Dhaka | 15234653 | 2025 |
| Dinajpur | 3276021 | 2021 |
| Dinajpur | 3315238 | 2022 |
| Dinajpur | 3352347 | 2023 |
| Dinajpur | 3389887 | 2024 |
| Dinajpur | 3427882 | 2025 |
| Faridpur | 2136303 | 2021 |
| Faridpur | 2162876 | 2022 |
| Faridpur | 2187101 | 2023 |
| Faridpur | 2211592 | 2024 |
| Faridpur | 2236365 | 2025 |
| Feni | 1629237 | 2021 |
| Feni | 1648896 | 2022 |
| Feni | 1667345 | 2023 |
| Feni | 1686025 | 2024 |
| Feni | 1704921 | 2025 |
| Gaibandha | 2531113 | 2021 |
| Gaibandha | 2562232 | 2022 |
| Gaibandha | 2590912 | 2023 |
| Gaibandha | 2619913 | 2024 |
| Gaibandha | 2649290 | 2025 |
| Gazipur | 5199379 | 2021 |
| Gazipur | 5263474 | 2022 |
| Gazipur | 5322387 | 2023 |
| Gazipur | 5381974 | 2024 |
| Gazipur | 5442314 | 2025 |
| Gopalganj | 1279197 | 2021 |
| Gopalganj | 1295053 | 2022 |
| Gopalganj | 1309556 | 2023 |
| Gopalganj | 1324227 | 2024 |
| Gopalganj | 1339055 | 2025 |
| Habiganj | 2329838 | 2021 |
| Habiganj | 2358886 | 2022 |
| Habiganj | 2385298 | 2023 |
| Habiganj | 2411998 | 2024 |
| Habiganj | 2439035 | 2025 |
| Jamalpur | 2468537 | 2021 |
| Jamalpur | 2499737 | 2022 |
| Jamalpur | 2527732 | 2023 |
| Jamalpur | 2556037 | 2024 |
| Jamalpur | 2584672 | 2025 |
| Jashore | 3037794 | 2021 |
| Jashore | 3076849 | 2022 |
| Jashore | 3111309 | 2023 |
| Jashore | 3146160 | 2024 |
| Jashore | 3181393 | 2025 |
| Jhalokati | 653202 | 2021 |
| Jhalokati | 661161 | 2022 |
| Jhalokati | 668565 | 2023 |
| Jhalokati | 676056 | 2024 |
| Jhalokati | 683625 | 2025 |
| Jhenaidah | 1981081 | 2021 |
| Jhenaidah | 2005849 | 2022 |
| Jhenaidah | 2028319 | 2023 |
| Jhenaidah | 2051032 | 2024 |
| Jhenaidah | 2074003 | 2025 |
| Joypurhat | 945069 | 2021 |
| Joypurhat | 956430 | 2022 |
| Joypurhat | 967137 | 2023 |
| Joypurhat | 977969 | 2024 |
| Joypurhat | 988927 | 2025 |
| Khagrachhari | 705859 | 2021 |
| Khagrachhari | 714119 | 2022 |
| Khagrachhari | 722118 | 2023 |
| Khagrachhari | 730208 | 2024 |
| Khagrachhari | 738383 | 2025 |
| Khulna | 2581453 | 2021 |
| Khulna | 2613385 | 2022 |
| Khulna | 2642642 | 2023 |
| Khulna | 2672240 | 2024 |
| Khulna | 2702181 | 2025 |
| Kishoreganj | 3226509 | 2021 |
| Kishoreganj | 3267630 | 2022 |
| Kishoreganj | 3304211 | 2023 |
| Kishoreganj | 3341196 | 2024 |
| Kishoreganj | 3378656 | 2025 |
| Kurigram | 2300915 | 2021 |
| Kurigram | 2329161 | 2022 |
| Kurigram | 2355247 | 2023 |
| Kurigram | 2381627 | 2024 |
| Kurigram | 2408300 | 2025 |
| Kushtia | 2122259 | 2021 |
| Kushtia | 2149692 | 2022 |
| Kushtia | 2173775 | 2023 |
| Kushtia | 2198105 | 2024 |
| Kushtia | 2222733 | 2025 |
| Lakshmipur | 1915076 | 2021 |
| Lakshmipur | 1938111 | 2022 |
| Lakshmipur | 1960818 | 2023 |
| Lakshmipur | 1983760 | 2024 |
| Lakshmipur | 2006963 | 2025 |
| Lalmonirhat | 1411137 | 2021 |
| Lalmonirhat | 1428406 | 2022 |
| Lalmonirhat | 1444396 | 2023 |
| Lalmonirhat | 1460586 | 2024 |
| Lalmonirhat | 1476939 | 2025 |
| Madaripur | 1277185 | 2021 |
| Madaripur | 1293027 | 2022 |
| Madaripur | 1307503 | 2023 |
| Madaripur | 1322146 | 2024 |
| Madaripur | 1336961 | 2025 |
| Magura | 1020706 | 2021 |
| Magura | 1033115 | 2022 |
| Magura | 1044689 | 2023 |
| Magura | 1056389 | 2024 |
| Magura | 1068217 | 2025 |
| Manikganj | 1539083 | 2021 |
| Manikganj | 1558024 | 2022 |
| Manikganj | 1575466 | 2023 |
| Manikganj | 1593104 | 2024 |
| Manikganj | 1610962 | 2025 |
| Maulvibazar | 2097156 | 2021 |
| Maulvibazar | 2123445 | 2022 |
| Maulvibazar | 2147204 | 2023 |
| Maulvibazar | 2171246 | 2024 |
| Maulvibazar | 2195594 | 2025 |
| Meherpur | 696819 | 2021 |
| Meherpur | 705356 | 2022 |
| Meherpur | 713248 | 2023 |
| Meherpur | 721239 | 2024 |
| Meherpur | 729322 | 2025 |
| Munshiganj | 1605495 | 2021 |
| Munshiganj | 1625418 | 2022 |
| Munshiganj | 1643627 | 2023 |
| Munshiganj | 1662022 | 2024 |
| Munshiganj | 1680646 | 2025 |
| Mymensingh | 5825393 | 2021 |
| Mymensingh | 5899052 | 2022 |
| Mymensingh | 5965097 | 2023 |
| Mymensingh | 6031858 | 2024 |
| Mymensingh | 6099488 | 2025 |
| Naogaon | 2750089 | 2021 |
| Naogaon | 2784598 | 2022 |
| Naogaon | 2815789 | 2023 |
| Naogaon | 2847297 | 2024 |
| Naogaon | 2879212 | 2025 |
| Narail | 779126 | 2021 |
| Narail | 788673 | 2022 |
| Narail | 797508 | 2023 |
| Narail | 806446 | 2024 |
| Narail | 815470 | 2025 |
| Narayanganj | 3861472 | 2021 |
| Narayanganj | 3909138 | 2022 |
| Narayanganj | 3952896 | 2023 |
| Narayanganj | 3997124 | 2024 |
| Narayanganj | 4041961 | 2025 |
| Narsingdi | 2553403 | 2021 |
| Narsingdi | 2584452 | 2022 |
| Narsingdi | 2613383 | 2023 |
| Narsingdi | 2642646 | 2024 |
| Narsingdi | 2672265 | 2025 |
| Natore | 1836836 | 2021 |
| Natore | 1859921 | 2022 |
| Natore | 1880745 | 2023 |
| Natore | 1901798 | 2024 |
| Natore | 1923116 | 2025 |
| Chapainawabganj | 1813327 | 2021 |
| Chapainawabganj | 1835527 | 2022 |
| Chapainawabganj | 1856082 | 2023 |
| Chapainawabganj | 1876867 | 2024 |
| Chapainawabganj | 1897894 | 2025 |
| Netrokona | 2295775 | 2021 |
| Netrokona | 2324856 | 2022 |
| Netrokona | 2350876 | 2023 |
| Netrokona | 2377186 | 2024 |
| Netrokona | 2403849 | 2025 |
| Nilphamari | 2067149 | 2021 |
| Nilphamari | 2092567 | 2022 |
| Nilphamari | 2115991 | 2023 |
| Nilphamari | 2139682 | 2024 |
| Nilphamari | 2163667 | 2025 |
| Noakhali | 3580941 | 2021 |
| Noakhali | 3625252 | 2022 |
| Noakhali | 3665855 | 2023 |
| Noakhali | 3706900 | 2024 |
| Noakhali | 3748429 | 2025 |
| Pabna | 2873730 | 2021 |
| Pabna | 2909622 | 2022 |
| Pabna | 2942203 | 2023 |
| Pabna | 2975169 | 2024 |
| Pabna | 3008484 | 2025 |
| Panchagarh | 1165705 | 2021 |
| Panchagarh | 1179843 | 2022 |
| Panchagarh | 1193056 | 2023 |
| Panchagarh | 1206413 | 2024 |
| Panchagarh | 1219931 | 2025 |
| Patuakhali | 1706532 | 2021 |
| Patuakhali | 1727254 | 2022 |
| Patuakhali | 1746597 | 2023 |
| Patuakhali | 1766168 | 2024 |
| Patuakhali | 1785942 | 2025 |
| Pirojpur | 1183729 | 2021 |
| Pirojpur | 1198193 | 2022 |
| Pirojpur | 1211608 | 2023 |
| Pirojpur | 1225173 | 2024 |
| Pirojpur | 1238904 | 2025 |
| Rajbari | 1175416 | 2021 |
| Rajbari | 1189821 | 2022 |
| Rajbari | 1203149 | 2023 |
| Rajbari | 1216628 | 2024 |
| Rajbari | 1230248 | 2025 |
| Rajshahi | 2879367 | 2021 |
| Rajshahi | 2915013 | 2022 |
| Rajshahi | 2947641 | 2023 |
| Rajshahi | 2980656 | 2024 |
| Rajshahi | 3014058 | 2025 |
| Rangamati | 639752 | 2021 |
| Rangamati | 647587 | 2022 |
| Rangamati | 654834 | 2023 |
| Rangamati | 662169 | 2024 |
| Rangamati | 669590 | 2025 |
| Rangpur | 3130844 | 2021 |
| Rangpur | 3169615 | 2022 |
| Rangpur | 3205106 | 2023 |
| Rangpur | 3240989 | 2024 |
| Rangpur | 3277311 | 2025 |
| Satkhira | 2170006 | 2021 |
| Satkhira | 2196581 | 2022 |
| Satkhira | 2221167 | 2023 |
| Satkhira | 2246029 | 2024 |
| Satkhira | 2271215 | 2025 |
| Shariatpur | 1278652 | 2021 |
| Shariatpur | 1294561 | 2022 |
| Shariatpur | 1309051 | 2023 |
| Shariatpur | 1323709 | 2024 |
| Shariatpur | 1338547 | 2025 |
| Sherpur | 1483318 | 2021 |
| Sherpur | 1501853 | 2022 |
| Sherpur | 1518676 | 2023 |
| Sherpur | 1535683 | 2024 |
| Sherpur | 1552882 | 2025 |
| Sirajganj | 3315527 | 2021 |
| Sirajganj | 3357708 | 2022 |
| Sirajganj | 3395326 | 2023 |
| Sirajganj | 3433350 | 2024 |
| Sirajganj | 3471795 | 2025 |
| Sunamganj | 2661895 | 2021 |
| Sunamganj | 2695495 | 2022 |
| Sunamganj | 2725668 | 2023 |
| Sunamganj | 2756196 | 2024 |
| Sunamganj | 2787081 | 2025 |
| Sylhet | 3808540 | 2021 |
| Sylhet | 3857037 | 2022 |
| Sylhet | 3900206 | 2023 |
| Sylhet | 3943828 | 2024 |
| Sylhet | 3988090 | 2025 |
| Tangail | 3987376 | 2021 |
| Tangail | 4037608 | 2022 |
| Tangail | 4082818 | 2023 |
| Tangail | 4128506 | 2024 |
| Tangail | 4174796 | 2025 |
| Thakurgaon | 1515026 | 2021 |
| Thakurgaon | 1533894 | 2022 |
| Thakurgaon | 1551068 | 2023 |
| Thakurgaon | 1568438 | 2024 |
| Thakurgaon | 1586012 | 2025 |

# Geograpical Data Used In Cluster Analysis

***Tab.S7*** Full geographic data used in this study

| District | Longitude | Latitude |
| --- | --- | --- |
| Bagerhat | 89.978 | 22.1435 |
| Bandarban | 92.2141 | 22.1567 |
| Barguna | 90.135 | 22.17 |
| Barishal | 90.3471 | 22.701 |
| Bhola | 90.7294 | 22.416 |
| Bogura | 89.3706 | 24.8467 |
| Brahmanbaria | 91.1056 | 23.9642 |
| Chandpur | 90.6666 | 23.224 |
| Chapainawabganj | 88.2462 | 24.6273 |
| Chattogram | 91.8344 | 22.4433 |
| Chuadanga | 88.8453 | 23.6311 |
| CoxsBazar | 92.1387 | 21.4509 |
| Cumilla | 91.1761 | 23.4681 |
| Dhaka | 90.2508 | 23.787 |
| Dinajpur | 88.6489 | 25.6269 |
| Faridpur | 89.8336 | 23.547 |
| Feni | 91.4 | 23.0274 |
| Gaibandha | 89.5122 | 25.2748 |
| Gazipur | 90.5613 | 24.0183 |
| Gopalganj | 89.8209 | 23.0126 |
| Habiganj | 91.4101 | 24.3085 |
| Jamalpur | 89.9312 | 24.919 |
| Jashore | 89.2044 | 23.1116 |
| Jhalokati | 90.1893 | 22.6406 |
| Jhenaidah | 89.1505 | 23.5524 |
| Joypurhat | 89.0333 | 25.0891 |
| Khagrachhari | 91.9915 | 23.1103 |
| Khulna | 89.4478 | 22.4709 |
| Kishoreganj | 90.8006 | 24.4371 |
| Kurigram | 89.6594 | 25.812 |
| Kushtia | 89.0995 | 23.9085 |
| Lakshmipur | 90.8339 | 22.9543 |
| Lalmonirhat | 89.259 | 25.9234 |
| Madaripur | 90.2145 | 23.1728 |
| Magura | 89.3858 | 23.4845 |
| Manikganj | 90.0081 | 23.8552 |
| Maulvibazar | 91.7746 | 24.4826 |
| Meherpur | 88.6316 | 23.786 |
| Munshiganj | 90.5244 | 23.5473 |
| Mymensingh | 90.4353 | 24.7568 |
| Naogaon | 88.7378 | 24.8101 |
| Narail | 89.5003 | 23.144 |
| Narayanganj | 90.602 | 23.688 |
| Narsingdi | 90.7852 | 23.9391 |
| Natore | 89.0177 | 24.4128 |
| Netrokona | 90.709 | 24.8533 |
| Nilphamari | 88.9173 | 25.9465 |
| Noakhali | 91.1043 | 22.8634 |
| Pabna | 89.2379 | 24.0062 |
| Panchagarh | 88.5613 | 26.3417 |
| Patuakhali | 90.3477 | 22.215 |
| Pirojpur | 89.9815 | 22.5823 |
| Rajbari | 89.6508 | 23.7191 |
| Rajshahi | 88.6042 | 24.372 |
| Rangamati | 92.1777 | 22.6556 |
| Rangpur | 89.2368 | 25.6498 |
| Satkhira | 89.075 | 22.5445 |
| Shariatpur | 90.3829 | 23.2107 |
| Sherpur | 90.0177 | 25.0209 |
| Sirajganj | 89.7218 | 24.3444 |
| Sunamganj | 91.3952 | 25.0288 |
| Sylhet | 91.9873 | 24.9192 |
| Tangail | 89.9171 | 24.2727 |
| Thakurgaon | 88.4367 | 25.9962 |

# Dengue Ord Gi* Values

***Tab.S8*** Gi* Values

| **District** | **Gi*_Raw_2021** | **p_value_Raw_2021** | **Gi*_Raw_2022** | **p_value_Raw_2022** | **Gi*_Raw_2023** | **p_value_Raw_2023** | **Gi*_Raw_2024** | **p_value_Raw_2024** | **Gi*_Raw_2025** | **p_value_Raw_2025** | **Gi*_Rate_2025** | **p_value_Rate_2025** |
| --- | --- | --- | --- | --- | --- | --- | --- | --- | --- | --- | --- | --- |
| Bagerhat | 0.010408 | 0.478302 | 0.030822 | 0.414833 | 0.014126 | 0.484601 | 0.018122 | 0.482966 | 0.023712 | 0.425791 | 0.046392 | 0.079169 |
| Bandarban | 0.007679 | 0.467478 | 0.022857 | 0.460005 | 0.019005 | 0.47232 | 0.019688 | 0.47135 | 0.027863 | 0.388004 | 0.018873 | 0.446518 |
| Barguna | 0.002397 | 0.44664 | 0.00438 | 0.43451 | 0.013464 | 0.478951 | 0.0121 | 0.472117 | 0.029502 | 0.373485 | 0.061848 | 0.019036 |
| Barisal | 0.002418 | 0.446736 | 0.003969 | 0.432095 | 0.013935 | 0.483118 | 0.010681 | 0.461717 | 0.018207 | 0.478733 | 0.040659 | 0.133334 |
| Bhola | 0.002744 | 0.448068 | 0.003124 | 0.427187 | 0.015936 | 0.49945 | 0.012817 | 0.477027 | 0.022381 | 0.439502 | 0.02934 | 0.272439 |
| Bogra | 0.000216 | 0.438043 | 0.000719 | 0.413643 | 0.001167 | 0.372407 | 0.003121 | 0.404431 | 0.002355 | 0.373958 | 0.003163 | 0.278827 |
| Brahmanbaria | 0.00334 | 0.450307 | 0.008577 | 0.458088 | 0.007426 | 0.425885 | 0.022205 | 0.452345 | 0.013248 | 0.475164 | 0.010315 | 0.399068 |
| Chandpur | 0.003024 | 0.44929 | 0.006718 | 0.447635 | 0.011706 | 0.463723 | 0.016606 | 0.494525 | 0.015102 | 0.492753 | 0.017582 | 0.46898 |
| Chapainawabganj | 0.001514 | 0.443113 | 0.000944 | 0.414732 | 0.001634 | 0.375916 | 0.005681 | 0.423218 | 0.002599 | 0.375594 | 0.002567 | 0.268852 |
| Chittagong | 0.001178 | 0.442857 | 0.002664 | 0.428785 | 0.005736 | 0.414302 | 0.005999 | 0.428622 | 0.009382 | 0.441196 | 0.017928 | 0.462794 |
| Chuadanga | 0.001145 | 0.441666 | 0.002918 | 0.425773 | 0.010356 | 0.451347 | 0.008091 | 0.441314 | 0.008295 | 0.428188 | 0.013705 | 0.46004 |
| Comilla | 0.001217 | 0.442675 | 0.003462 | 0.430846 | 0.007444 | 0.426754 | 0.010799 | 0.463441 | 0.008892 | 0.434898 | 0.008896 | 0.374426 |
| Coxs Bazar | 0.009398 | 0.474405 | 0.033819 | 0.39823 | 0.021495 | 0.451094 | 0.026204 | 0.423297 | 0.029488 | 0.376199 | 0.027606 | 0.298999 |
| Dhaka | 0.040722 | 0.270499 | 0.012914 | 0.472447 | 0.02392 | 0.405894 | 0.031939 | 0.197495 | 0.027597 | 0.28085 | 0.018308 | 0.45633 |
| Dinajpur | 0.000482 | 0.439139 | 0.000308 | 0.411145 | 0.001086 | 0.37147 | 0.001064 | 0.389941 | 0.000498 | 0.35737 | 0.00092 | 0.245317 |
| Faridpur | 0.10208 | 0.195602 | 0.07095 | 0.215166 | 0.053904 | 0.204287 | 0.060735 | 0.20091 | 0.048381 | 0.21917 | 0.024701 | 0.341781 |
| Feni | 0.010299 | 0.477886 | 0.030637 | 0.415853 | 0.019814 | 0.465349 | 0.033146 | 0.371721 | 0.0241 | 0.422622 | 0.009065 | 0.377947 |
| Gaibandha | 0.000856 | 0.440529 | 0.001122 | 0.415617 | 0.002111 | 0.380012 | 0.00328 | 0.405509 | 0.003239 | 0.381372 | 0.003461 | 0.282514 |
| Gazipur | 0.141898 | 0.113524 | 0.10454 | 0.102767 | 0.090118 | 0.053213 | 0.087247 | 0.091624 | 0.072568 | 0.097573 | 0.021789 | 0.394868 |
| Gopalganj | 0.001531 | 0.443187 | 0.006213 | 0.444559 | 0.014556 | 0.48844 | 0.012417 | 0.473901 | 0.020801 | 0.453137 | 0.040375 | 0.128159 |
| Habiganj | 0.000585 | 0.43946 | 0.001448 | 0.417466 | 0.002039 | 0.379313 | 0.003369 | 0.406153 | 0.006117 | 0.407981 | 0.005098 | 0.309157 |
| Jamalpur | 0.001775 | 0.444184 | 0.010562 | 0.46943 | 0.028048 | 0.393635 | 0.006618 | 0.430737 | 0.006757 | 0.414475 | 0.004358 | 0.298893 |
| Jessore | 0.010602 | 0.47916 | 0.029443 | 0.423285 | 0.011738 | 0.463944 | 0.013354 | 0.48117 | 0.015339 | 0.494985 | 0.023067 | 0.371505 |
| Jhalokathi | 0.001021 | 0.441197 | 0.006369 | 0.445483 | 0.016757 | 0.492191 | 0.020737 | 0.463363 | 0.035854 | 0.31987 | 0.070577 | 0.005966 |
| Jhenaidah | 0.001594 | 0.443491 | 0.004869 | 0.436931 | 0.011256 | 0.459661 | 0.006027 | 0.426278 | 0.007334 | 0.419775 | 0.010328 | 0.399908 |
| Joypurhat | 0.00051 | 0.43917 | 0.001475 | 0.417628 | 0.001762 | 0.376991 | 0.002905 | 0.402722 | 0.00274 | 0.376945 | 0.00231 | 0.265646 |
| Khagrachhari | 0.009134 | 0.473234 | 0.031665 | 0.409825 | 0.020864 | 0.455958 | 0.022175 | 0.452483 | 0.026411 | 0.400967 | 0.012082 | 0.431491 |
| Khulna | 0.002029 | 0.447757 | 0.006997 | 0.454707 | 0.007972 | 0.431723 | 0.006972 | 0.435638 | 0.008075 | 0.429454 | 0.014948 | 0.483453 |
| Kishoreganj | 0.007021 | 0.464954 | 0.011802 | 0.476655 | 0.031386 | 0.365788 | 0.009536 | 0.452533 | 0.01398 | 0.482238 | 0.009181 | 0.38013 |
| Kurigram | 0.000961 | 0.440939 | 0.000376 | 0.411469 | 0.001908 | 0.378326 | 0.00276 | 0.401605 | 0.002276 | 0.372628 | 0.002573 | 0.268764 |
| Kushtia | 0.000857 | 0.440628 | 0.002707 | 0.425109 | 0.005435 | 0.409539 | 0.004539 | 0.41567 | 0.003314 | 0.38386 | 0.006121 | 0.329393 |
| Lakshmipur | 0.001966 | 0.444942 | 0.003461 | 0.42903 | 0.012369 | 0.469609 | 0.013375 | 0.481166 | 0.021345 | 0.448356 | 0.025123 | 0.336049 |
| Lalmonirhat | 0.000919 | 0.440809 | 0.000656 | 0.412998 | 0.00179 | 0.377282 | 0.000815 | 0.387405 | 0.000525 | 0.356904 | 0.000542 | 0.238695 |
| Madaripur | 0.00131 | 0.442501 | 0.003681 | 0.430594 | 0.012351 | 0.469315 | 0.011468 | 0.467047 | 0.013074 | 0.473761 | 0.017966 | 0.462585 |
| Magura | 0.001947 | 0.444861 | 0.005998 | 0.443436 | 0.013619 | 0.480329 | 0.008862 | 0.447149 | 0.007398 | 0.419983 | 0.013937 | 0.464601 |
| Manikganj | 0.135402 | 0.117857 | 0.094416 | 0.129378 | 0.06284 | 0.157172 | 0.079059 | 0.120628 | 0.059887 | 0.152626 | 0.015308 | 0.489909 |
| Maulvibazar | 0 | 0.437166 | 0.000526 | 0.412297 | 0.00087 | 0.369452 | 0.001343 | 0.391225 | 0.005389 | 0.401088 | 0.004355 | 0.29669 |
| Meherpur | 0.000868 | 0.440611 | 0.003532 | 0.429271 | 0.00776 | 0.428947 | 0.006527 | 0.429971 | 0.007792 | 0.423655 | 0.010708 | 0.406723 |
| Munshiganj | 0.118398 | 0.153758 | 0.085362 | 0.15818 | 0.057605 | 0.178512 | 0.078733 | 0.117695 | 0.058147 | 0.156848 | 0.023193 | 0.367956 |
| Mymensingh | 0.007634 | 0.467569 | 0.003515 | 0.433545 | 0.008022 | 0.435975 | 0.010112 | 0.457162 | 0.015249 | 0.494198 | 0.010892 | 0.41007 |
| Naogaon | 0.000755 | 0.440135 | 0.001359 | 0.417074 | 0.001643 | 0.37603 | 0.003323 | 0.405838 | 0.002694 | 0.376555 | 0.00328 | 0.279995 |
| Narail | 0.009491 | 0.474683 | 0.027161 | 0.435582 | 0.01392 | 0.482845 | 0.013691 | 0.483579 | 0.012001 | 0.463504 | 0.014059 | 0.467555 |
| Narayanganj | 0.142822 | 0.102944 | 0.097981 | 0.119748 | 0.063337 | 0.147447 | 0.089783 | 0.085903 | 0.070305 | 0.101349 | 0.020434 | 0.417776 |
| Narsingdi | 0.01027 | 0.477772 | 0.006335 | 0.44531 | 0.008927 | 0.439503 | 0.015474 | 0.497042 | 0.022311 | 0.439721 | 0.014762 | 0.479832 |
| Natore | 0.000953 | 0.440917 | 0.004376 | 0.434135 | 0.00374 | 0.39388 | 0.005283 | 0.420466 | 0.004465 | 0.392937 | 0.005064 | 0.309351 |
| Netrakona | 0.003823 | 0.452239 | 0.023651 | 0.455303 | 0.061205 | 0.157478 | 0.008987 | 0.448091 | 0.01323 | 0.474903 | 0.008339 | 0.364124 |
| Nilphamari | 0.00108 | 0.441427 | 0.000618 | 0.412807 | 0.001647 | 0.376042 | 0.002485 | 0.399605 | 0.001546 | 0.366057 | 0.001454 | 0.251979 |
| Noakhali | 0.006616 | 0.463258 | 0.017528 | 0.490469 | 0.01778 | 0.483206 | 0.021156 | 0.46018 | 0.019006 | 0.470302 | 0.014735 | 0.479061 |
| Pabna | 0.001382 | 0.442615 | 0.003191 | 0.428285 | 0.012807 | 0.472889 | 0.008616 | 0.445287 | 0.009488 | 0.439439 | 0.015948 | 0.49861 |
| Panchagarh | 0.000567 | 0.439389 | 0.000551 | 0.412409 | 0.000912 | 0.369847 | 0.002934 | 0.402932 | 0.00176 | 0.367964 | 0.001628 | 0.254543 |
| Patuakhali | 0.00143 | 0.443289 | 0.005105 | 0.438443 | 0.013695 | 0.48113 | 0.019775 | 0.4709 | 0.026186 | 0.405474 | 0.037361 | 0.16797 |
| Pirojpur | 0.000674 | 0.439866 | 0.003659 | 0.430483 | 0.009409 | 0.444124 | 0.012171 | 0.472287 | 0.016062 | 0.498277 | 0.036438 | 0.147661 |
| Rajbari | 0.00169 | 0.443842 | 0.006253 | 0.444835 | 0.016612 | 0.493536 | 0.010656 | 0.460517 | 0.008873 | 0.433853 | 0.014589 | 0.476566 |
| Rajshahi | 0.000503 | 0.439319 | 0.002781 | 0.425023 | 0.00388 | 0.395209 | 0.003986 | 0.41153 | 0.005014 | 0.398049 | 0.006739 | 0.336797 |
| Rangamati | 0.006281 | 0.461928 | 0.022679 | 0.460859 | 0.014913 | 0.491505 | 0.018325 | 0.481431 | 0.0203 | 0.457955 | 0.022814 | 0.375146 |
| Rangpur | 0.000477 | 0.439184 | 0.000435 | 0.411848 | 0.001099 | 0.371829 | 0.001982 | 0.395996 | 0.001149 | 0.362605 | 0.001092 | 0.246836 |
| Satkhira | 0.026757 | 0.456853 | 0.073192 | 0.204408 | 0.016829 | 0.491566 | 0.028889 | 0.402777 | 0.03261 | 0.345532 | 0.034393 | 0.197743 |
| Shariatpur | 0.002506 | 0.44714 | 0.004705 | 0.436009 | 0.013446 | 0.478684 | 0.014961 | 0.493112 | 0.019637 | 0.464627 | 0.026641 | 0.313804 |
| Sherpur | 0.004697 | 0.455695 | 0.033385 | 0.400299 | 0.090792 | 0.048653 | 0.012518 | 0.474649 | 0.01617 | 0.497176 | 0.010323 | 0.39893 |
| Sirajganj | 0.001451 | 0.44287 | 0.003982 | 0.431867 | 0.008637 | 0.436214 | 0.009612 | 0.452844 | 0.009384 | 0.438416 | 0.012332 | 0.434839 |
| Sunamganj | 0.000816 | 0.440378 | 0.001557 | 0.418089 | 0.002072 | 0.379569 | 0.003821 | 0.409396 | 0.007146 | 0.417404 | 0.006329 | 0.329073 |
| Sylhet | 6.8E-05 | 0.437429 | 0.000255 | 0.410822 | 0.000215 | 0.36415 | 0.000592 | 0.385888 | 0.000679 | 0.359612 | 0.000814 | 0.244577 |
| Tangail | 0.142953 | 0.103263 | 0.103753 | 0.102364 | 0.093994 | 0.042409 | 0.083685 | 0.102846 | 0.073176 | 0.088852 | 0.026121 | 0.31969 |
| Thakurgaon | 0.000663 | 0.439769 | 0.000649 | 0.412964 | 0.001099 | 0.371399 | 0.003951 | 0.410451 | 0.002462 | 0.374307 | 0.002225 | 0.263502 |

# Full Sensitivity Analysis

To ensure the robustness of our findings, we conducted comprehensive sensitivity analyses across both spatial autocorrelation (Getis-Ord Gi*) and space-time scan statistics (SaTScan™), varying key methodological parameters.

## 1. Local Spatial Autocorrelation (Gi*) Sensitivity

We re-ran the 2025 rate-adjusted Getis-Ord Gi* analysis using alternative **spatial weights matrices**, **population bases**, and **2025 case data handling** (Table [X]). All scenarios used queen or k-nearest neighbor (KNN) contiguity with row-standardization.

***Tab.S9*** Gi* Sensitivity summary

| **Scenario** | **Weights** | **Population** | **2025 Cases** | **Significant Hotspots (p<0.05)** | **Key Coastal Hotspots** |
| --- | --- | --- | --- | --- | --- |
| **Primary** | Queen | Projected 2025 | Annualized | 2 | Barguna, Jhalokathi |
| Sensitivity 1 | Queen | Projected 2025 | Annualized | 2 | Barguna, Jhalokathi |
| Sensitivity 2 | Rook | Projected 2025 | Annualized | 2 | Barguna, Jhalokathi |
| Sensitivity 3 | KNN4 | Projected 2025 | Annualized | 5 | Bagerhat, Barguna, Barishal, Jhalokathi, Patuakhali |
| Sensitivity 4 | KNN6 | Projected 2025 | Annualized | 4 | Bagerhat, Barguna, Patuakhali, Pirojpur |
| Sensitivity 5 | Queen | Projected 2025 | 310-day raw (non-annualized) | 2 | Barguna, Jhalokathi |
| Sensitivity 6 | Queen | 2022 Census (no projection) | Annualized | 2 | Barguna, Jhalokathi |

***Supplementary Figure 1.*** *Sensitivity of 2025 Getis-Ord Gi hotspots to data handling and population assumptions.*

** All panels show statistically significant (p < 0.05) high-rate hotspots (red) using queen contiguity weights and rate-adjusted incidence per 100,000. (A) Non-annualized 310-day raw 2025 cases (Jan–Nov 6). (B) 2022 Census population (no projection). (C) Primary analysis: annualized 2025 cases + projected 2025 population. The core hotspots in Barguna and Jhalokathi remain unchanged across all scenarios, confirming robustness. Basemap: OpenStreetMap contributors (CC-BY-SA).*

The 2025 Getis-Ord Gi* hotspot analysis remained robust across methodological variations: using non-annualized 310-day raw case data (January 1–November 6, 2025) or the 2022 census population without projection yielded the identical pair of significant high-rate hotspots in Barguna and Jhalokathi (p <0.05) matching the primary result with annualized cases and projected 2025 population under queen contiguity weights.

***Supplementary Figure 2*.** Sensitivity of 2025 Getis-Ord Gi hotspots to different weight metrics

Queen and Rook contiguity consistently identified Barguna and Jhalokathi as hotspots. KNN4 and KNN6 expanded the cluster to include Bagerhat, Barishal, Patuakhali, and Pirojpur, reflecting greater spatial smoothing but confirming the southern coastal emergence. Non-annualized 310-day data and 2022 census population yielded identical core hotspots, indicating minimal sensitivity to partial-year reporting or population projection.

## 2. Space-Time Cluster Detection (SaTScan) Sensitivity

Prospective space-time scan statistics are instrumental in identifying emerging disease clusters. However, the sensitivity of these methods to parameter selection, particularly the maximum spatial and temporal scanning windows, necessitates rigorous validation. Our primary analysis employed a liberal scanning window (spatial: 50% of at-risk population; temporal: 50% of study period). To quantify the robustness of our findings, we conducted a comprehensive sensitivity analysis by systematically constraining these parameters. Our objective was to evaluate the stability of identified dengue fever clusters in Bangladesh under varying analytical constraints, confirming the validity of the reported spatiotemporal risk patterns.

***Supplementary Figure 2*.** Sensitivity of SaTScan parameters

We executed four separate prospective space-time analyses using a discrete Poisson model, holding the study period (2021–2025), population data, and inference settings (999 replicates, p<0·001) constant. The models were defined by their maximum scanning windows:

1. **Base Model:** Liberal spatial (50% population) and temporal (50% period) windows.
2. **Spatially Restricted Model:** Constrained spatial (30% population), liberal temporal (50% period) windows.
3. **Temporally Restricted Model:** Liberal spatial (50% population), constrained temporal (30% period) windows.
4. **Fully Restricted Model:** Constrained spatial (30% population) and temporal (30% period) windows.

Cluster robustness was assessed based on the consistent identification of core geographic areas with congruent risk direction (high/low) and magnitude across models.

The sensitivity analysis demonstrates that the principal conclusions are not artefacts of a single model specification. The hyperendemic core in Dhaka and the emerging high-risk zone in the Barishal Division are exceptionally robust.

**1. Stability of High-Risk Clusters (Relative Risk > 1)**

***Tab.S10*** (SaTScan) Sensitivity Analysis of High-Risk Clusters

| **Cluster Description** | **Base Model** | **Spatially Restricted** | **Temporally Restricted** | **Fully Restricted** | **Interpretation** |
| --- | --- | --- | --- | --- | --- |
| **Dhaka & Manikganj** (Urban Hyperendemic Core) | **Detected** RR: 3·13 Period: 2024-25 LLR: 31777·1 | **Detected** RR: 3·13 Period: 2024-25 LLR: 31777·1 | **Detected** RR: 2·34 Period: 2025 LLR: 7830·3 | **Detected** RR: 2·34 Period: 2025 LLR: 7830·3 | **Extremely Robust.** The core cluster is invariant. Temporal restriction refines the signal to the peak year (2025), reflected in a lower, yet highly significant, LLR. |
| **Barishal Division** (Jhalokati, Barishal, Pirojpur, Patuakhali) | **Detected** RR: 2·51 Period: 2025 LLR: 3761·7 | **Detected** RR: 2·51 Period: 2025 LLR: 3761·7 | **Detected** RR: 2·51 Period: 2025 LLR: 3761·7 | **Detected** RR: 2·51 Period: 2025 LLR: 3761·7 | **Extremely Robust.** Perfect consistency across all models in location, population, and elevated risk, indicating a sharp, recent, and stable signal. |
| **Khulna District** (Focused High-Risk Area) | **Detected** RR: 2·11 Period: 2024-25 LLR: 1879·0 | **Detected** RR: 2·11 Period: 2024-25 LLR: 1879·0 | Not Detected | Not Detected | **Temporally Sensitive.** This cluster is only identified when the analysis allows for longer temporal durations (2-year periods), suggesting a persistent but less intense signal than Clusters 2 and 4. |

**2. Stability of Low-Risk Clusters (Relative Risk < 1)**

***Tab.S11*** (SaTScan) Sensitivity Analysis of Low-Risk Clusters

| **Cluster Description** | **Base Model** | **Spatially Restricted** | **Temporally Restricted** | **Fully Restricted** | **Interpretation** |
| --- | --- | --- | --- | --- | --- |
| **Northern Region** (Broad Low-Incidence Zone) | **Detected** RR: 0·16 Districts: 23 LLR: 52921·0 | **Detected** RR: 0·12 Districts: 18 LLR: 44340·9 | **Detected** RR: 0·15 Districts: 23 LLR: 27090·6 | **Detected** RR: 0·08 Districts: 18 LLR: 23975·9 | **Spatially Robust.** A large, stable low-risk zone. Spatial restriction logically narrows the cluster from 23 to 18 districts, refining its core area and increasing the observed/expected difference. |
| **Northeastern Region** (Sylhet and surrounding divisions) | **Detected** RR: 0·44 Districts: 17 LLR: 14197·6 | **Detected** RR: 0·38 Districts: 15 LLR: 20724·8 | **Detected** RR: 0·43 Districts: 16 LLR: 7278·7 | **Detected** RR: 0·39 Districts: 15 LLR: 8954·1 | **Spatially Robust.** A consistent low-risk region. Its boundaries are slightly refined under spatial restrictions, and its temporal signal strengthens in the final year. |
| **Satkhira District** (Isolated Low-Risk District) | Not Detected | **Detected** RR: 0·28 LLR: 1236·0 | Not Detected | **Detected** RR: 0·28 LLR: 621·7 | **Context-Dependent.** This isolated low-risk district is identified only when the spatial window is restricted, preventing it from being absorbed into a larger regional cluster. |

**3. Synthesis of Sensitivity Findings**

*Figure 1. Conceptual Map of Cluster Robustness* would be included here, showing:

- **Stable High-Risk Cores (Red):** Dhaka and Barishal, persistent across all models.
- **Variable High-Risk Area (Orange):** Khulna, present only in models with longer temporal windows.
- **Stable Low-Risk Regions (Blue):** Northern and Northeastern zones, consistently identified with modifiable boundaries.
- **Variable Low-Risk Area (Light Blue):** Satkhira, detected only under spatial restriction.

The Log-Likelihood Ratio (LLR), a measure of how much more likely a cluster is under the alternative hypothesis of an outbreak versus the null hypothesis of random distribution, remained exceptionally high and significant (p<0·001) for all detected clusters across all models, confirming that these are not marginal findings.

This systematic sensitivity analysis demonstrates that the core findings of a dual dengue geography in Bangladesh are robust. The hyperendemicity of the Dhaka core and the emergence of a high-risk zone in the Barishal Division are statistically significant phenomena observable under a wide range of analytical assumptions. The consistent identification of these clusters, despite varying constraints, strongly suggests they represent true biological and epidemiological phenomena rather than statistical artefacts.

The analysis also provides valuable insights into the nature of these clusters:

- The **Dhaka cluster's** stability under spatial restriction confirms its intense, localized nature, while its persistence under temporal restriction highlights sustained transmission.
- The **Barishal cluster's** perfect consistency across all models indicates a sharp, recent, and geographically focused emergence, warranting urgent public health attention.
- The **low-risk northern and northeastern clusters** represent vast, stable regions of lower endemicity, though their exact contours can be refined with spatial parameters.

The case of Khulna and Satkhira illustrates how sensitivity analysis reveals nuanced patterns: Khulna represents a more prolonged, lower-intensity risk, while Satkhira is a true low-risk outlier only visible when not subsumed into a broader region.

In conclusion, this rigorous assessment validates our primary model and strengthens the evidence for targeting the identified stable high-risk zones for intensive intervention, while also clarifying the spatiotemporal character of dengue risk across Bangladesh. Public health policy can be formulated with confidence based on these robust, cross-validated results.

***Supplementary Figure 2*.** Sensitivity of 2025 Getis-Ord Gi hotspots to data handling and population assumptions

***Supplementary Figure 2*.** Sensitivity of 2025 Getis-Ord Gi hotspots to data handling and population assumptions

***Supplementary Figure 2*.** Sensitivity of 2025 Getis-Ord Gi hotspots to data handling and population assumptions
