# Supplementary material for: Urban Hyperendemicity and Emerging Coastal Per‐Capita Risk of Dengue in Bangladesh, 2021–2025: A Nationwide Spatiotemporal Ecological Analysis
Source: J Trop Med. 2026 Aug 7;2026:3012361. doi: 10.1155/jotm/3012361 (PMC13451933; doi:10.1155/jotm/3012361)
Supplement: Supplementary file 1 [file JOTM-2026-3012361-s001.zip › Supplementary Tab.S12 STROBE Statement_2.docx]

**STROBE Statement – Checklist of Items for the Manuscript**

**Title:** *Dual Geography of Dengue: Urban Hyperendemicity and Emerging Coastal Risk in Bangladesh, 2021–2025* **Study design:** Retrospective spatiotemporal ecological analysis

| **Item No** | **STROBE Recommendation** | **Location in Manuscript** | **Details / Compliance** |
| --- | --- | --- | --- |
| **1(a)** | Indicate the study’s design with a commonly used term in the title or the abstract | Title; Abstract (Design) | “Retrospective ecological spatiotemporal analysis” |
| **1(b)** | Provide in the abstract an informative and balanced summary of what was done and what was found | Abstract | Structured: Objectives, Design, Setting, Participants, Outcomes, Results, Conclusions |
| **2** | Explain the scientific background and rationale for the investigation being reported | Introduction (paras 1–3) | Global dengue burden, historical outbreaks in Bangladesh, geographic expansion, prior study gaps |
| **3** | State specific objectives, including any prespecified hypotheses | Introduction (final para); Abstract (Objectives) | “Analyse retrospective dengue fever incidence clustering… providing population-adjusted insights” |
| **4** | Present key elements of study design early in the paper | Methods → Study Design and Setting | Retrospective ecological study; 64 districts; 2021–2025; two analytic frameworks |
| **5** | Describe the setting, locations, and relevant dates, including periods of recruitment, exposure, follow-up, and data collection | Methods → Study Design and Setting; Data Sources | Nationwide Bangladesh; 1 Jan 2021 – 31 Dec 2025; weekly/daily surveillance data |
| **6(a)** | (a) Cohort study – Give the eligibility criteria, and the sources and methods of selection of participants… | N/A (ecological) – aggregate data | Methods → Data Sources: “Confirmed and probable dengue cases per national definitions” from DGHS |
| **6(b)** | (b) Cohort study – For matched studies… | N/A | — |
| **7** | Clearly define all outcomes, exposures, predictors, potential confounders, and effect modifiers. Give diagnostic criteria, if applicable | Methods → Spatial Statistical Analyses; Incidence Rate Calculation | Outcomes: incidence/100,000, clusters (Gi*, SaTScan); population adjustment; equations provided |
| **8** | For each variable of interest, give sources of data and details of methods of assessment (measurement). Describe comparability of assessment methods if there is more than one group | Methods → Data Sources | Dengue: DGHS weekly/daily reports (2025a, 2025b); Population: BBS Census 2022 + projections; Geography: BBS centroids |
| **9** | Describe any efforts to address potential sources of bias | Methods → Population Data; Analyses | Population adjustment (exponential & uniform growth); annualisation of 2025 partial data; Monte Carlo inference |
| **10** | Explain how the study size was arrived at | Results → Table 1 | Fixed: all 64 districts, 5 full years (637,626 cases) |
| **11** | Explain how quantitative variables were handled in the analyses. If applicable, describe which groupings were chosen and why | Methods → Incidence Rate; Spatial Analyses | Continuous incidence; Poisson model; Gi* (p<0.05); LLR/RR equations |
| **12(a)** | Describe all statistical methods, including those used to control for confounding | Methods → Spatial Statistical Analyses | Moran’s I, Getis-Ord Gi* (Queen weights), SaTScan Discrete Poisson; 999 Monte Carlo; population adjustment |
| **12(b)** | Describe any methods used to examine subgroups and interactions | Results → Hotspot overlap; Figures 1–2 | Raw vs rate-adjusted hotspots; no overlap reported |
| **12(c)** | Explain how missing data were addressed | Implicit | Complete surveillance coverage; no missing districts |
| **12(d)** | Cohort study – If applicable, explain how loss to follow-up was addressed | N/A | — |
| **12(e)** | Describe any sensitivity analyses | Included – see Methods, Sensitivity Analysis for Annualization; also Supplementary Figures S1–S3 and Supplementary Tables S9–S11 | — |
| **13(a)** | Report numbers of individuals at each stage of study… | N/A (aggregate) | — |
| **13(b)** | Give characteristics of study participants… | Results → Table 1 | Total cases, population, national rate |
| **13(c)** | Indicate number of participants with missing data for each variable of interest | None | Complete dataset |
| **14(a)** | Report numbers of outcome events or summary measures over time | Results → Tables 1, 2, 5, 6 | Annual raw & rate; space-time clusters |
| **14(b)** | Give unadjusted estimates and, if applicable, confounder-adjusted estimates… | All results population-adjusted | Rate-adjusted Gi*, SaTScan Poisson model |
| **14(c)** | Report category boundaries when continuous variables were categorized | Results → Figure 2 | Quantile map (72.5–329.4/100,000) |
| **15** | Report other analyses done – e.g. analyses of subgroups and interactions, and sensitivity analyses | Results → Hotspot overlap; Discussion | Raw vs rate dissociation; alignment with prior studies |
| **16(a)** | Give unadjusted estimates and, if applicable, confounder-adjusted estimates… | Results → Tables 2–6 | O/E, RR, LLR, p-values |
| **16(b)** | Report results with confidence intervals or p-values | All tables | p-values; LLR; Monte Carlo p=0.001 |
| **16(c)** | Discuss multiplicity of analyses | Methods → SaTScan | 999 Monte Carlo replications |
| **17** | Summarise key results with reference to study objectives | Abstract (Results); Discussion | Dual geography; urban vs coastal risk |
| **18** | Discuss limitations of the study, taking into account sources of potential bias or imprecision… | Included – see Discussion, Limitations subsection | Included – see Discussion, Limitations subsection |
| **19** | Discuss the generalisability (external validity) of the study results | Discussion | National coverage; implications for tropical urbanising settings |
| **20** | Give a cautious overall interpretation considering objectives, limitations, multiplicity… | Discussion | “Urgent need for adaptable, targeted interventions” |
| **21** | Give the source of funding and the role of the funders… | None | No funding |
| **22** | Give information on ethics approval or waiver | N/A (public aggregate data) | — |

**All 22 applicable STROBE items are addressed.** **Manuscript complies fully with STROBE guidelines for observational studies (v4, 2007).** **Prepared:** 10 November 2025, Dhaka, Bangladesh
